# The Prebiotic Potential of Inulin-Type Fructans: A Systematic Review

**DOI:** 10.1093/advances/nmab119

**Published:** 2021-10-27

**Authors:** Riley L Hughes, David A Alvarado, Kelly S Swanson, Hannah D Holscher

**Affiliations:** Department of Food Science and Human Nutrition, University of Illinois at Urbana-Champaign, Champaign, IL, USA; Department of Food Science and Human Nutrition, University of Illinois at Urbana-Champaign, Champaign, IL, USA; Division of Nutrition Sciences, University of Illinois at Urbana-Champaign, Champaign, IL, USA; Department of Animal Sciences, University of Illinois at Urbana-Champaign, Champaign, IL, USA; Department of Food Science and Human Nutrition, University of Illinois at Urbana-Champaign, Champaign, IL, USA; Division of Nutrition Sciences, University of Illinois at Urbana-Champaign, Champaign, IL, USA

**Keywords:** inulin-type fructans, fructooligosaccharides, oligofructose, prebiotic, gut microbiota, human health, clinical studies

## Abstract

Inulin-type fructans (ITF), including short-chain fructooligosaccharides (scFOS), oligofructose, and inulin, are commonly used fibers that are widely regarded as prebiotic for their ability to be selectively utilized by the intestinal microbiota to confer a health benefit. However, to our knowledge the literature thus far lacks a thorough discussion of the evidence from human clinical trials for the prebiotic effect of ITF, including beneficial effects on intestinal microbiota composition and intestinal and extraintestinal processes (e.g., glucose homeostasis, lipids, mineral absorption and bone health, appetite and satiety, inflammation and immune function, and body composition). Additionally, there has been a lack of discussion regarding aspects such as the effect of ITF chain length on its intestinal and extraintestinal effects. The overall objective of this systematic review was to summarize the prebiotic potential of ITF based on the results of human clinical trials in healthy adult populations. Evidence from studies included in the current review suggest that ITF have a prebiotic effect on the intestinal microbiota, promoting the abundances of *Bifidobacterium, Lactobacillus*, and *Faecalibacterium prausnitzii*. Beneficial health effects reported following ITF intake include improved intestinal barrier function, improved laxation, increased insulin sensitivity, decreased triglycerides and an improved lipid profile, increased absorption of calcium and magnesium, and increased satiety. Although there is some evidence for differing effects of ITF based on chain length, the lack of direct comparisons and detailed descriptions of physicochemical properties limits the ability to draw conclusions from human clinical studies. Future research should focus on elucidating the mechanisms by which the intestinal microbiota mediates or modifies the effects of ITF on human health and the contribution of individual factors such as age and metabolic health to the movement toward personalization of prebiotic applications.

## Teaser

**Statement of Significance**: The current review provides a comprehensive synthesis of results from clinical studies in healthy human adults on the prebiotic effects of ITF on the intestinal microbiota and health, with consideration for effects of ITF chain length and individual factors such as baseline intestinal microbiota composition.

## Introduction

Diet-related chronic diseases such as obesity, metabolic syndrome, type 2 diabetes, and cardiovascular disease (CVD) are becoming increasingly prevalent and are related to insufficient fiber intake (1). Less than 10% of Americans consume the recommended amount of fiber (14 g per 1000 kcal) (2). Fiber is not digested and thus is available for metabolism by intestinal microbiota (3). The intestinal microbiota is thought to contribute to the effects of diet on human health (4). Therefore, the observed benefits of dietary fiber may be partially mediated by the intestinal microbiota (3).

Prebiotics are defined by the International Scientific Association for Probiotics and Prebiotics (ISAPP) as “a substrate that is selectively utilized by host microorganisms conferring a health benefit” (5). The criterion of selective utilization distinguishes prebiotics from other substances or compounds that may broadly affect the gastrointestinal microbiota (5). The guiding principle of this criterion is that a limited number of microbial groups are affected and that the microbial groups and metabolites affected are linked to a beneficial health effect (5).

Some, though not all, dietary fibers may be classified as prebiotics, though most prebiotics can be classified as dietary fibers (5, 6). Currently, only inulin-type fructans (ITF), galactooligosaccharides (7), and lactulose are accepted by ISAPP as prebiotics (5). ITF is a general term referring to all β(21→) linear fructans and includes native inulin [degree of polymerization (DP) 2–60], short-chain fructooligosaccharides (scFOS; DP 2–4), and oligofructose (DP <10) (8–10). Physical separation is required to purify long-chain inulin (8, 9). Oligofructose is a shorter-chain inulin extracted from plants and can also be produced by partial enzymatic hydrolysis of inulin (10). scFOS can be manufactured from sucrose and fructose by an enzymatic process or may also be extracted from plants (10). Native inulin and oligofructose are found in artichokes, asparagus, bananas, chicory root, garlic, onions, leeks, and wheat (3). However, most commercially available ITF are synthesized from sucrose or extracted from chicory roots, Jerusalem artichoke, and agave (11). Commercially, ITF are added to a variety of food products. The longer chain length of inulin reduces its solubility but gives it a creamy texture, lending itself to function as a fat replacement in spreads, baked goods, dairy products, frozen desserts, and dressings (11). The shorter chain length of scFOS increases their solubility (11). This lends scFOS properties similar to those of sugar, though they provide ∼30–50% of the sweetness of table sugar and are commonly used in cereals, fruit yogurts, frozen desserts, and cookies (11).

The chain length and branching of ITF subtypes may also influence the fermentation characteristics within the intestines (12–14), thus impacting their effects on the intestinal microbiota and/or health outcomes. ITF with shorter DPs (e.g., DP <10) are fermented more rapidly in the distal small intestine or proximal colon, whereas longer-chain inulin is fermented more slowly (15, 16). A mixture of inulin and oligofructose may thus be more effective at stimulating the production of bioactive metabolites (e.g., SCFAs) throughout the intestinal tract. A previous review suggested that the prebiotic effects of different ITF types on bifidobacterial populations were comparable based on the use of a “prebiotic index” defined as “the increase in bifidobacteria expressed as the absolute number (*N*) of ‘new’ colony-forming units/g of feces divided by the daily dose (in g) of ITF ingested” (9). However, the efficacy of different types of ITF may also depend on the outcome of interest (e.g., bifidobacterial population, mineral absorption, immune response, etc.) (13, 17).

The prevalence of ITF in everyday food items, both naturally and via supplementation, makes it important to assess the potential contribution of these compounds to the intestinal microbiota and human health. To review and substantiate the health benefits of isolated or synthetic fibers, the US FDA requires evidence of beneficial effects from studies in healthy human populations (10). Therefore, this criterion was used in the current review to assess the prebiotic potential of ITF within the scope of the ISAPP prebiotic definition. Additionally, to our knowledge no previous reviews have documented the effect of chain length of ITF on the intestinal microbiota or health outcomes in human trials. Therefore, our objective was to establish the prebiotic potential of ITF, using results from clinical trials in healthy adults to explore health benefits correlated with ITF intake. Additionally, we aimed to summarize available evidence for the effects of ITF chain length on their prebiotic effects.

## Methods

We searched PubMed, Web of Science, and the Cumulative Index to Nursing and Allied Health Literature (CINAHL) in February 2021 for full-length English articles published in peer-reviewed journals with no date restriction. Briefly, we divided search terms into 2 categories: *1*) ITF (e.g., inulin, oligofructose, fructans, fructooligosaccharides) AND *2*) microbiota OR health outcomes (e.g., microbiota, microbiome, *Bifidobacterium*, glucose, lipids, inflammation, satiety, blood pressure, mineral, laxation). Eligibility criteria were similar to those used by the FDA to determine the physiological effects of inulin (10). These criteria included the following: *1*) the use of an appropriate control group (i.e., nonbioactive control or placebo), *2*) the use of appropriate statistical methods, *3*) the use of ITF in isolation (i.e., not in combination with energy restriction or other bioactive dietary components), *4*) studies conducted in healthy human populations, and *5*) the intervention being of sufficient length of time to capture the gastrointestinal component (≥1 wk) (10). This last criterion ensured that there was a sufficient amount of time for the intervention to induce and stabilize changes in the intestinal microbiome, which could then be evaluated as potential contributors to observed health benefits. The current review also included only studies in healthy adults (≥18 y of age). Studies with multiple treatment arms were included if ≥1 arm met the inclusion criteria. The full search strategy and details for the current review were registered in PROSPERO (CRD42021240531). We followed the recommended Preferred Reporting Items for Systematic Reviews and Meta-Analyses (PRISMA) guidelines for article selection.

The title and abstract of nonduplicate articles were screened based on the above criteria. The remaining publications were reviewed in full by RLH and DAA, and reference lists of eligible articles were screened for additional publications not identified in the initial searches. HDH and KSS resolved any discrepancies in study inclusion or exclusion. RLH and DAA extracted the data from studies meeting the selection criteria, including information on the study population [age, BMI (in kg/m^2^), population characteristics], intervention (study design, duration), the ITF supplement (type, dose, chain length), and effects on the intestinal microbiota and health outcomes compared with the control group.

## Results and Discussion

### Study selection and characteristics

**FIGURE 1:**
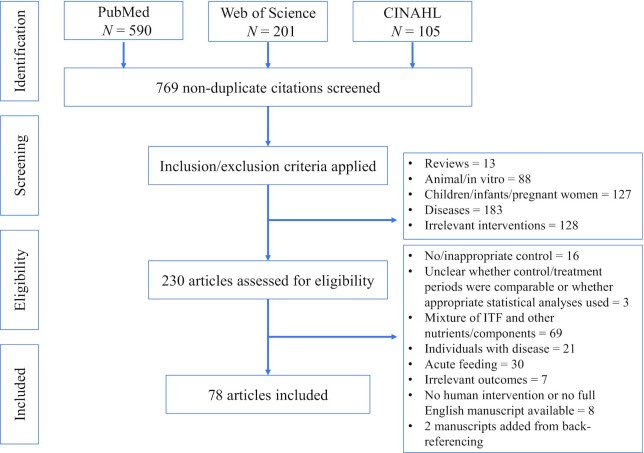
PRISMA flow diagram of article search and selection process. The literature search was conducted in February of 2021 and included all full-length articles in English published in peer-reviewed journals with no date restriction recorded in PubMed, Web of Science, and CINAHL. PRISMA, Preferred Reporting Items for Systematic Reviews and Meta-Analyses; CINAHL, Cumulative Index to Nursing and Allied Health Literature.

Overall, 78 publications were included (**Figure 1**). Of these 78 publications, 45 reported results on the fecal microbiota and related metabolites (e.g., SCFAs, bile acids). Thirty-six publications included results on gastrointestinal physiology, including intestinal permeability, transit time, and tolerance. Eighteen publications included results on cardiovascular health, 15 on glucose homeostasis, 10 on mineral absorption and bone health, 13 on appetite and satiety, 10 on body composition and energy balance, and 5 on inflammation. To ensure that interventions were of sufficient duration to capture the changes occurring in the gastrointestinal tract, 1 wk was used as the minimum duration, though studies ranged up to 24 mo. Doses ranged from 2.5 to 50 g/d. Inulin-oligofructose blends or scFOS with an average DP ≤10 were the most commonly tested forms of ITF. The following sections explore the potential prebiotic effects of ITF on the intestinal microbiome, including composition and functional aspects such as metabolite production, and on human health outcomes or biomarkers with consideration for the effects of ITF chain length and baseline fecal microbiota composition.

### Effects of ITF on the fecal microbiota and metabolites

The most commonly reported finding from studies investigating fecal microbiota composition was an increase in or higher abundance of *Bifidobacterium* spp. (∼1.8–3.8-fold) following ITF consumption (33/35 studies, **Table 1**) (18–62). The remaining 2 studies reported no effect of ITF on *Bifidobacterium* (28, 54). No studies reported a decrease in *Bifidobacterium*. Other effects on the fecal microbiota composition were more variable. For example, 4 studies reported an increase in (relative to baseline) or higher (relative to control) microbial diversity or richness (23, 29, 54, 57), 3 reported a decrease in or lower richness (26, 30, 31), and 4 reported no effect or mixed effects when comparing doses (21, 25, 43, 46, 48). Five studies reported an increase in or higher abundance of *Lactobacillus* (∼1.83–5.88-fold) (33, 36, 46, 54, 57, 59), whereas 5 reported no effect (22–24, 28, 43). Specific stimulations of *Bifidobacterium* and *Lactobacillus* are considered prebiotic effects as these taxa have been associated with a range of health benefits (5). Other prebiotic effects may include SCFA-producing taxa such as *Faecalibacterium prausnitzii* (5). Five studies reported an increase in *F. prausnitzii* or *Ruminococcus* (36, 39, 42, 49, 57), both of which are butyrate-producing taxa. One study reported a lower abundance of genus *Ruminococcus* compared with the control (30) and 3 studies reported either no change or varying response patterns among participants depending on factors such as habitual dietary fiber intake or changes in Bacteroidetes abundance (31, 39, 43). Abundances of members of the genus *Bacteroides* increased or were higher compared with baseline or placebo in 2 studies (30, 42) but decreased in 3 studies (33, 36, 43) and was not changed in 4 studies (22, 23, 28, 46). Of the included studies, clostridia abundance decreased following ITF intake in 4 studies (30, 40, 43, 55), increased in abundance in 1 study (36), and were unchanged in 2 studies (28, 54).

**TABLE 1:** ITF effects on the gut microbiome^1^

Ref	Study design	Comparator	ITF type (trade name, manufacturer)	DP, range (mean)	ITF dosage, g/d	Intervention duration, wk	Washout duration, wk	N (*n*in ITF group)	Study population characteristics	Analytical method(s)	Results
(18)	MC, OLS	Glucose	scFOS (Raftilose P95 Beneo-Orafti)	2–7	0, 5, 15	1	1	24	Healthy adult men age 19–28 y, BMI 21.7 ± 1.9	SCFAs (GC)	↑ Breath H_2_ significant only in 15 g/d FOS↔ Fecal SCFAs, pH
(19)	R, DB, PC, P	Maltodextrin	scFOS (Actilight 950P, Beghin-Meiji)	2–4	5	4	NA	79 (41)	IBS and rectal sensitivity in adults age 41.7 y, BMI 22.73	qPCR (*Eubacteria, Bif, Lactobacillus, Enterobacteriaceae, Roseburia, E. rectale, F. prausnitzii*)	↑ *Bif*
(20)	R, DB, SC, PC, P	Maltodextrin	Inulin (Beneo-Orafti HSI)	NA	8	4	NA	36 (18)	Healthy adults with GI symptoms, 20–70 y	qPCR (*Bif*, total bacteria)	↑ *Bif*
(21)	R, DB, PC, P	Saccharose	scFOS (Actilight, Eridania-Beghin Say)	2–4	12.5	1.7	1.7	20	Healthy adults age 22–39 y	Plate culture (total anaerobes, *Bif*) Enzyme activity assays	↑ *Bif*, β-fructosidase activity↔ Fecal bile acids, neutral sterols, total anaerobes, pH, nitroreductase, azoreductase, β-glucuronidase
(22)	R, DB, PC, P	Sucrose/Maltodextrin	Inulin (Fibruline Instant, Cosucra)	2–60 (10)	5	4	NA	39 (20)	Healthy adults age 33.9 y	Plate culture (total anaerobes, *Bif, Lactobacillus, Bacteroides, Enterobacteria*) SCFAs (GC)	↑ *Bif* (not modified by baseline abundance)↔ *Lactobacillus, Enterobacteria, Bacteroides*, total anaerobes, SCFAs, β-galactosidase, nitrate reductase, nitroreductase, azoreductase ↓ β-glucuronidase β-glucuronidase ∼*Bif* (–)
(23)	R, DR, PC, P	Sucrose/Maltodextrin	scFOS (Actilight, Beghin Meiji)	2–4	0, 2.5, 5, 7.5, 10	1	NA	40 (32)	Healthy adults age 29 ± 1.3 y	Plate culture (total anaerobes, *Bif, Lactobacillus, Bacteroides, Enterobacteria*)	↑ *Bif* (all doses vs. placebo), total anaerobes (10 g vs. placebo)↔ *Bacteroides, Lactobacillus, Enterobacteria*, pH
(24)	R, DB, DR, PC, CF	Sucrose/Maltodextrin	scFOS (Actilight, Beghin Meiji)Long-chain inulin (Raftiline HP, Orafti)	scFOS 2–4HP: 5–60 (10)	Phase 1: 10, phase 2: 2.5, 5, 7.5, 10 scFOS only	1	NA	Phase 1: 64 (16), phase 2: 136 (32)	Healthy adults age 30 y	Plate culture (total anaerobes, *Bif, Lactobacillus, Bacteroides, Enterobacteria*)	↑ *Bif* (scFOS, not long-chain inulin)↔ *Bacteroides, Lactobacillus, Enterobacteria*, pH
(25)	R, DR, PC, P	Saccharose	scFOS (Actilight, Beghin Meiji)	2–4	2.5, 5, 10, 20	1	NA	40 (32)	Healthy adults age 29.6 y	Plate culture (total anaerobes, *Bif*)	↑ *Bif* (5, 10, 20 g), total anaerobes (20 g)↔ pH
(26)	PC, CF, SB	Cereal (rice)	Inulin (Fibruline Instant, Cosucra)	2–60 (10)	9	4	4	12	Healthy adult men age 23.3 ± 0.5 y, BMI 25.7 ± 1.2	Plate culture (total anaerobes, total facultative anaerobes, *Bif, Bacteroidaceae, Clostridia, Coliforms*)	↑ *Bif*↔ Fecal bile acids, fecal SCFAs, pH↓ Total anaerobes
(27)	R, DB, PC, CF	No ITF	Inulin (Beneo HP, DKSH/Orafti)	≥23	10	1	2	21	Adults with overweight/obesity, age 60 y, BMI 25–40	SCFAs (GC-MS)	↔ Fasting and postprandial SCFAs (acetate, propionate, butyrate)
(28)	R, DB, PC, P, LS	Water	Inulin (Fibruline Instant, Cosucra)	2–60 (10)	10	4	NA	51 (?)	Healthy adults age 30.9 ± 12.8 y, BMI 22.7 ± 2.2	qPCR (*Bif, Lactobacillus, Bacteroides, C. difficile, Enterococci*)	↔ *Bif, Lactobacillus, Bacteroides, Enterococci, C. difficile*
(39)	R, P	Low nonstarch polysaccharide diet	Chicory inulin (Agro-industries,Recherches et Developpements Society)	NA	2 wk @ progressive intake, 12 d @ 50	3.7	NA	9	Healthy adult men age 21.5 ± 2.5, BMI <25	Microbial mass (purine-base content)	↑ Microbial mass excretion, N content of microbial mass
(30)	R, DB, PC, CF	Cellulose	Inulin (Inulin HP, Beneo-Orafti)	≥23	20	6	≥3 (mean 6.3)	12	Nondiabetic adults with overweight/obesity, age 60 ± 1 y, BMI 29.8 ± 0.9	16S rRNA gene sequencingSCFAs (NMR)	↑ *Actinobacteria* (*B. faecale*), *Bacteroides caccae, Anaerostipes* hadrus↓ *Clostridia, Blautia, Ruminococcus faecis, Oscillibacter spp*, microbial diversity↔ Fecal or serum SCFAs
(31)	R, DB, PC, CF	Maltodextrin	Inulin–oligofructose (Synergy 1, Beneo-Orafti)	Oligofructose 2–8 (4)Inulin 10–60 (24)	15	3	2	30	Healthy adults age 28.2 ± 5.1 y, BMI 24.5 ± 3.0	Plate culture, T-RFLP, 16S rRNA gene sequencing, metagenomicsSCFAs (GC)	↓Microbial richnessResponse pattern 1: ↑ *Bif, Faecalibacterium, Lachnospiraceae*; ↓ *Bacteroidaceae*Response pattern 2: ↑ Bacteroidetes, *Bif*; ↓ *Faecalibacterium, Lachnospiraceae*↔ Metagenome, fecal SCFAs
(32)	R, DB, PC, CF	Maltodextrin	Inulin–oligofructose (Synergy 1, Beneo-Orafti)	Oligofructose: 2–8 (4)Inulin: 10–60 (24)	15	3	2	30	Healthy adults age 28.1 ± 5.1 y, BMI 24.2 ± 3.0	qPCR (total bacteria, *Bif, Eubacteria*)SCFAs (GC)	↑ *Bif*, total fecal SCFAs (propionate and butyrate at expense of acetate and BCFAs)
(33)	R, DB, PC, CF	Maltodextrin	VLCI (Bayer Cropscience AG) + Inulin (Beneo HP, Beneo-Orafti)	VLCI 50–103, HP ≥23	10	3	3	31	Healthy adults age 25 y, BMI 20–30	FISH (total bacteria, *Bif, Lactobacillus, Bacteroides*-*Prevotella, Clostridium, Ruminococcus, Eubacterium*)SCFAs (HPLC)	↑ *Bif, Lactobacillus*-*Enterococcus*↓ *Bacteroides*-*Prevotella*↔ *E. coli, E. rectale-C. coccoides, Clostridium*, fecal SCFAs
(34)	R, SB, PC, CF	No ITF	Inulin (Inulin HP, Beneo-Orafti)	≥23	22.4	1	1	13	Healthy adults age 23 ± 4 y, BMI 22.1 ± 1.6	SCFAs (GC)	↔ Serum SCFAs
(35)	R, SB, PC, P	Cellulose + Maltodextrin	Oligofructose (Beneo P95, Beneo-Orafti)	2–7	30	6	NA	22 (12)	Healthy adults with overweight and obesity age 20–49 y, BMI 30.33	SCFAs (GC)	↑ H_2_, serum acetate, propionate, butyrate
(36)	R, DB, PC, P	Maltodextrin	Inulin–oligofructose (Synergy 1, Beneo-Orafti)	Oligofructose: 2–8 (4) Inulin: 10–60 (24)	16	12	NA	30 (15)	Adult women with obesity age 47.5 y, BMI 35.85	qPCR (*Bif, Lactobacillus*)HITChip	↑ *Bif, F. prausnitzii, Lactobacillus, Clostridium* clusters IV and XVI↓ *Bacteroides, Propionibacterium*
(37)	R, CF	No ITF	Inulin–oligofructose (Beneo, DKSH/Orafti)	NA	10	3	0	12	Healthy adults age 38 ± 2.43 y, BMI 25.0 ± 1.09	qPCR (total bacteria, *Bif*)	↑ *Bif*
(38)	R, DB, PC, CF	Maltodextrin + aspartame	scFOS (Actilight P950, Beghin Meiji)	2–4	10.6	8	4	30	Adults with mild hypercholesterolemia (TC 5.17–7.76 mmol/L and TG <3.45 mmol/L), age 45.5 ± 9.9 y, BMI 26.6 ± 2.2	SCFAs (GC)	↔ Fasting plasma acetate
(39)	R, DB, PC, CF	Glucose	Inulin–oligofructose (Synergy 1, Beneo-Orafti)	Oligofructose: 2–8 (4)Inulin: 10–60 (24)	16	3	3	34	Healthy adults with low [<18 (F) or 22 (M) g/d) or high [≥25 (F) or 30 (M) g/d] dietary fiber intake (LDF or HDF), age 37.4 y, BMI 23.2	16S rRNA gene sequencingqPCR (*Bif*)SCFAs (GC)	↑ LDF: *Bif*; HDF: *Bif* and *Faecalibacterium*↓ HDF: *Coprococcus, Dorea, Ruminococcus*↔ α-diversity, fecal SCFAs
(40)	SGD	Habitual diet	ITF-rich vegetables (artichoke, garlic, salsify, shallot, leeks, scorzonera, onion, celery root)	NA	∼15	2	3	26	Healthy adults age 21.84 ± 0.39 y, BMI 22.29 ± 0.32	16S rRNA gene sequencing	↑ *Bif*↓ Clostridiales
(41)	R, DB, PC, CF	Placebo	Inulin (BIOAGAVE, Ingredion)	25–34	0, 5, 7.5	3	1	29	Healthy adults age 27 ± 4.1 y, BMI 24.4 ± 2.26	16S rRNA gene sequencingSCFAs (GC)	↑ Actinobacteria (5 g/d), *Bif* (7.5 g/d)↓ *Desulfovibrio*↔ Fecal SCFAsTotal dietary fiber ∼ fecal butyrate (+), *Bif* (+, trend), *Desulfovibrio* (–)
(42)	R, DB, PC, CF	Maltodextrin	scFOS	NA	16	1.4	3	20	Adults with IBS (diarrhea predominant or mixed) age 34.6 y, BMI 26.9	16S rRNA gene sequencingSCFAs (GC)	↑ *Bif*, Actinobacteria, *F. prausnitzii, Bacteroides*↓ Proteobacteria, *Mycoplasma hominis*↔ Fecal SCFAs
(43)	R, DB, PC, P	Cereal mixture	Artichoke inulin (Liven GmbH)Chicory inulin (Fibruline Instant, Cosucra)	5–12	1 wk @ 7.7, 2 wk @ 15.4	3	NA	45 (15)	Healthy adults age 23.5 + 2.3 y, BMI 22.4 ± 2.7 (F) 23.4 ± 1.4 (M)	FISH (total bacteria, *Clostridia*-*Eubacteria, Bacteroides*-*Prevotella, F. prausnitzii, Bif*)Plate culture (*Enterobacteriaceae, Lactobacillus, Enterococcus, Clostridium perfringens*, yeasts)SCFAs (GC)	↑ *Bif*↓ *Bacteroides*/*Prevotella, Clostridium*↔ Total bacteria, *Faecalibacterium, Lactobacillus, Enterococcus, C. perfringens*, yeasts, *Atopobium*, fecal SCFAs
(44)	DB, DR, PC, MCF	Maltodextrin	Inulin (Frutafit IQ, Sensus)	9–10	0, 5, 8	2	1	30	Healthy adults age 26.5 ± 3.1 y, BMI 20–30	FISH (*Bif, Bacteroides*-*Prevotella, Lactobacillus*-*Enterococcus, Clostridium perfringens*-*histolyticum*)	↑ *Bif* (higher % of volunteers responded to 8 g/d dose; baseline *Bif* abundance inversely correlated with increase)
(45)	CF	No inulin	Inulin (Fibruline, Cosucra)	2–50 (9)	22–34 (25% of energy from CHO)	9.14	4.9	8	Healthy adults age 26–53 y, BMI 22.8 ± 4.4 (F) 25.8 ± 1.6 (M)	FISH (total bacteria, *Bif*)SCFAs (GC)	↑ *Bif*↔ Fecal SCFAs
(46)	P	No ITF	Inulin–oligofructose (Raftiline HP + Raftilose P95, Beneo-Orafti)	HP: 5–60 (10)P95: 2–7	15	2	NA	29 (14)	Adults undergoing colonoscopy, age 35–72 y	Plate culture (fatty acid profile)	↑ *Bif, Lactobacillus*↔ Total anaerobes, *Bacteroides, Clostridium*
(47)	R, DB, PC, P	Maltodextrin	Inulin–oligofructose (Synergy 1, Beneo-Orafti)	Oligofructose: 2–8 (4)Inulin: 10–60 (24)	8	4	NA	43 (22)	Healthy adults age 45–63 y, BMI 25.36	FISH (total bacteria, *Bif*)	↑ *Bif*
(48)	R, DB, PC, CF	Maltodextrin	Inulin (Fibruline Instant, Cosucra)	2–60 (10)	20	4	2	32	Adult women with low iron (PF < 25μg/L), 18-40 y, BMI 21.5 ± 2.2	qPCR (total bacteria, *Bif*)SCFAs (HPLC)	↑ *Bif*, fecal lactate↓ Fecal pH↔ Fecal SCFAs, total bacteria
(49)	R, CF	No ITF	Inulin–oligofructose (Beneo, DKSH/Orafti)	NA	10	2.3	0	12	Healthy adults age 38 ± 2.43 y, BMI 25.0 ± 1.09	qPCR (total bacteria, *Bif, Bacteroides, Clostridia, Roseburia, Eubacterium, F. prausnitzii, Ruminococcus*, BCoAT)	↑ *F. prausnitzii, Bif. adolescenti*s, *Bif. bifidum*↔ Fecal SCFAs, pH, BCoAT
(50)	R, DB, DR, PC, CF	Placebo	ITF	NA	3 or 7	4	4	48	Healthy adults age 30.85 y, BMI 23.98	16S rRNA gene sequencingSCFAs (GC)	↑ *Bif* (7 g/d dose, 3 g/d dose not significant until targeted analysis w/qPCR) ↓ *Lachnospira, Oscillospira*↔ Fecal SCFAs, α-diversity, β-diversity
(51)	R, DB, PC, P	Placebo	Inulin (4 g) + Oligofructose (12 g)	NA	2 wk @ 8, 10 wk @ 16	12	NA	96 (26)	Adults with overweight/obesity, 39.8 y, BMI 31.5	16S rRNA gene sequencing	↑ *Bif*
(52)	R, DB, PC, P	Maltodextrin	Inulin–oligofructose (Synergy 1, Beneo-Orafti)	Oligofructose: 2–8 (4)Inulin: 10–60 (24)	16	12	NA	30 (15)	Adult women with obesity, 47.5 y, BMI 35.85	qPCR (*Bif*)SCFAs (GC)	↑ *Bif. longum, Bif. pseudocatenulatum, Bif. adolescentis*↓ Total fecal SCFAs, acetate, propionate
(53)	R, DB, PC, CF	Maltodextrin	scFOS (Raftilose P95, Beneo-Orafti)	2–7	25–30	2	2	12	Healthy adults age 21.4 ± 2 y, BMI 20.75	SCFAs (GC)	↑ Fecal acetate %↓ Fecal butyrate %↔ Fecal mucin-type oligosaccharide excretion
(54)	R, DB, PC, CF	Corn syrup	Inulin (Frutafit, Sensus)	9 (2–60)	20	3	0	12	Healthy adult men, 27–49 y, BMI < 32	Plate culture (total anaerobes, lactic acid bacteria, *Bif, Clostridium, Enterobacteria*)SCFAs (GC)	↑ Total anaerobes, *Lactobacillus*↓ Fecal ammonia, β-glucuronidase activity↔ *Bif, Clostridium, Enterobacteriaceae* (trending decrease)
(55)	SC, R, DB, PC, P	Maltodextrin	Oligofructose (Orafti P95, Beneo-Orafti)	2–8	14	1	NA	37 (19)	Healthy adults age 25 y, BMI 23	16S rRNA gene sequencingSCFAs (GC-MS)Metabolomics (LC-HRMS)	↑ Actinobacteria (*Bif*), Betaproteobacteria, Deltaproteobacteria (*Bilophila*), breath H_2_↓ *Clostridia* (*Lachnospiraceae, Anaerostipes, Blautia*), *Erysipelotrichi*↔ Fecal SCFAs, breath CH_4_
(56)	R, DB, PC, P	Sucrose	scFOS (NutraFlora, GTC Nutrition)	3–5	3	4	NA	62 (15)	Healthy adults age 25.3 y	SCFAs (GC)	↓ Fecal ammonia, isovalerate↔ Fecal SCFAS, pH
(57)	R, DB, DR, PC, P	Maltodextrin	scFOS (Fossence, Tata Chemicals Limited)	3–5	0, 2.5, 5, 10	12.85	NA	80 (20)	Healthy adults age 23–44 y, BMI 24 ± 3.2	16S rRNA gene sequencing	↑ *Bif, Lactobacillus*, diversity, butyrate-taxa (*Faecalibacterium, Ruminococcus, Oscillospira*)
(58)	DB, PC, CF	No ITF	Inulin (Cosucra)	NA	2.5	3	2	15	Healthy adults	Culture (*Enterobacteria, Lactobacillus, Enterococcus, Bif*)qPCR-DGGE (total bacteria)FISH (*Bif, Colinsella*)	↑ *Bif, C. aerofaciens*
(59)	R, DB, PC, CF	Sucrose	scFOS (Raftilose P95, Beneo-Orafti)	2–7	20	2	2	34	Healthy adult men, 27.7 ± 1.7 y, BMI 23.2 ± 0.5	qPCR (*Bif, Lactobacillus, E. coli*)	↑ *Bif, Lactobacillus*, lactic acid, fecal mucin excretion
(60)	DB, PC, CF	Maltodextrin	Inulin (Raftiline HP, Beneo-Orafti)	25	8	2	0	9	Healthy adults age 20–55 y	FISH (total bacteria, *Bif, Bacteroides, Clostridium, Lactobacillus*-*Enterococcus*)	↑ *Bif* (baseline *Bif* abundance inversely correlated with increase)↔ Total bacteria, *Bacteroides, Clostridium, Lactobacillus*-*Enterococcus*
(61)	R, DB, CF, LS	Basal diet	InulinscFOS	NA	15	3	0	12	Healthy adult men, 23+3 y, BMI 23	SCFAs (HPLC)Bile acids (GC)	↑ Fecal acetate and valerate (inulin), breath H_2_ (FOS)↓ Fecal deoxycholic acid and β-glucuronidase activity (inulin and FOS)↔ Other fecal bile acids or SCFAs
(62)	R, DB, DR, PC, CF	Sucrose	Oligofructose (Fructalose L92, Sensus)	2–10	1 wk @ 15, 1 wk @ 30	2	2	19	Healthy adults age 46.9 y, BMI 24.4	SCFAs and BCFAs (GC-MS)	↓ Fecal isovalerate, p-cresol↔ Fecal SCFAs, isobutyrate, total BCFAs

1 BCFA, branched chain fatty acid; BCoAT, butyryl-CoA acetate CoA-transferase; *Bif, Bifidobacterium*; C, cholesterol; CF, crossover feeding; CH_4_, methane; CHO, carbohydrate; DR, dose ranging; DB, double-blinded; DGGE, denaturing gradient gel electrophoresis; DP, degree of polymerization; FOS, fructooligosaccharide; FISH, fluorescent in situ hybridization; H_2_, hydrogen; HDF, high dietary fiber; HITChip, human intestinal tract chip; IBS, irritable bowel syndrome; ITF, inulin-type fructans; MCF, multiple crossover feeding; LC-HRMS, liquid chromatography-high resolution mass spectrometry; LDF, Low dietary fiber; LS, Latin square; NA, not applicable; OLS, orthogonal Latin-square; P, parallel; PC, placebo-controlled; Pr, prospective; R, randomized; Ref, reference; SB, single-blinded; SC, single-center; scFOS, short-chain fructooligosaccharides; SGD, single-group design; TB, triple blind; TC, total cholesterol; TG, triglyceride; T-RFLP, terminal restriction fragment length polymorphism; ˜ indicates a correlation; the parentheses indicate a positive (+) or negative (−) association.

The physiological effects and health benefits that result from the selective utilization of prebiotics rely on or may be explained by changes in microbial abundances (e.g., microbial components) as well as changes in microbiota functionality (e.g., gene expression) or microbial metabolites (5). Therefore, changes in both microbiota composition as well as metabolic function and metabolite production are relevant to prebiotic functionality. Only 2 studies assessed microbial function via metagenomics (31) or enzyme activity assays (21). Metagenomic analysis reported no effect of ITF consumption (31). However, enzyme activity assays revealed an increase in β-fructosidase activity, which degrades glycosidic bonds present in ITF and is prevalent in *Bifidobacterium* (21). Three studies also demonstrated a decrease in or lower β-glucuronidase activity (22, 54, 61), which was inversely associated with *Bifidobacterium* in 1 study (22). No changes were reported in bacterial enzyme activity of azoreductase, nitroreductase, or nitrate reductase (63).

The principal end products of microbial fermentation of nondigestible carbohydrates are SCFAs, primarily acetate, propionate, and butyrate (5). SCFAs play important roles in intestinal health as well as extraintestinal metabolic effects, including glucose homeostasis, lipid metabolism, the immune system, and appetite (64). However, despite the observed bifidogenic effect of ITF, only 1 study reported an increase in both *Bifidobacterium* and total fecal SCFAs (32). In that study, the concentration of propionate and butyrate increased by 0.07% and 3.0%, respectively, at the expense of acetate (2.2% decrease) and branched-chain fatty acids (BCFAs; 1.8% decrease) after 15 g/d of an inulin–oligofructose blend. Another study reported higher concentrations of serum acetate, propionate, and butyrate after 30 g/d oligofructose compared with control but did not measure the fecal microbiota composition (35). Conversely, 2 studies reported higher concentrations of fecal acetate and either lower or no difference in butyrate and other SCFAs (53, 61). Most studies reported no effect of ITF on SCFAs in feces (18, 22, 26, 30, 31, 41, 43, 48, 49, 55, 56, 62) or in blood (27, 30, 34, 38). Therefore, effects on SCFA production in humans consuming 3–30 g/d ITF for 1–12 wk are equivocal. It should be noted that because SCFAs and other fermentative metabolites are rapidly absorbed by the intestine, fecal samples are a poor measure of production and/or microbial activity, which may contribute to these contradictory findings (65).

In addition to direct metabolites of ITF degradation, ITF intake may indirectly impact the production of other metabolites such as bile acids and proteolytic metabolites via modulation of the intestinal microbiota composition or preferential metabolism of ITF. The intestinal microbiota impacts bile acid metabolism, with resultant impacts on host metabolic processes, including lipid metabolism and glucose homeostasis (66). One study reported that ITF intake (15 g/d inulin or scFOS) resulted in lower fecal concentrations of the secondary bile acid deoxycholic acid (61). However, 2 other studies reported no effect of ITF on fecal bile acids (21, 26). ITF consumption decreased or led to lower concentrations of the proteolytic metabolites fecal ammonia (54, 56), isovalerate (56, 62), and p-cresol (62), though isobutyrate and total BCFAs were not affected (62). Despite production of SCFAs—mainly propionate—from protein, proteolytic metabolism by the intestinal microbiota generally has a negative association with health, as toxic compounds can be produced from this process that increase inflammation, intestinal permeability, and risk of diseases such as colorectal cancer, obesity, and diabetes (67–69). Therefore, it may be the balance between saccharolytic and proteolytic metabolism and the resulting balance between production of potentially detrimental byproducts and the body's ability to assimilate, transform, or detoxify these byproducts that determines the effect on health outcomes (67, 69). Whereas different microbes vary in their metabolic functions and substrate preference, fiber is the preferred fuel source for the intestinal microbiota and increased fiber intake decreases proteolytic metabolism (70).

The prebiotic effects of ITF on the intestinal microbiota and resultant metabolites may be affected by plant source, chain length, dose, and individuals’ baseline fiber intake or intestinal microbiota composition, particularly the abundance of *Bifidobacterium* (8, 9, 71). Three studies directly compared different types of ITF as a function of plant source (e.g., Jerusalem artichoke compared with chicory) (43) or chain length (e.g., short-chain compared with long-chain) (24, 61). Both chicory- and artichoke-derived inulin had a bifidogenic effect with no statistical differences between treatments (43). One of the studies comparing inulin and scFOS did not provide information about the source of these supplements, so chain length could not be assessed (61). However, that study did report differential effects on fermentation metabolites, including higher fecal concentrations of acetate and valerate with inulin only and higher breath H_2_ with scFOS only (15 g/d). Moreover, both inulin and scFOS were associated with lower fecal deoxycholic acid concentrations and β-glucuronidase activity compared with control, as measured by enzymatic assay (61). Bouhnik et al. also compared ITF subtypes of different chain lengths (10 g/d) (24). In that study, scFOS (DP 2–4) had a bifidogenic effect, whereas long-chain inulin (DP 5–60, mean: 10) did not (24). Other studies testing long-chain inulin, however, have reported bifidogenic effects (26, 30, 33, 41, 44, 45, 48), though the 2 studies mentioned previously that reported no effect on *Bifidobacterium* tested long-chain inulin (28, 54).

Two studies reported an inverse correlation between baseline abundance of *Bifidobacterium* and increases in *Bifidobacterium* populations in response to ITF (44, 60). Holscher et al. also reported that total fiber intake (i.e., habitual fiber intake plus 0, 5, or 7.5 g/d ITF) was positively correlated with fecal butyrate and tended to be positively associated with *Bifidobacterium* (41).

### Prebiotic effects of ITF on human health outcomes

#### Gastrointestinal physiology

Measures of gastrointestinal physiology included were intestinal permeability and associated outcomes (fecal water cytotoxicity), transit time and stool characteristics (fecal score/composition and frequency), H_2_ gas production, and tolerance (abdominal pain, bloating, burping, flatulence, nausea, reflux, and rumbling) (**Table 2**) (18, 20, 27, 29, 32–35, 40, 42, 43, 45–47, 50, 51, 53–56, 59, 61, 62, 72–84).

**TABLE 2:** ITF effects on gastrointestinal health in healthy adults^1^

Ref.	Study design	Comparator	ITF type	DP, range (mean)	ITF dose, g/d	Intervention duration (wk)	Washout duration, wk	N (*n*in ITF group)	Study population characteristics	Results
(18)	MC, OLS	Glucose	FOS (Raftilose P95)	2–7	0, 5, 15	1	1	24	Healthy adult men age 19–28 y, BMI 21.7 ± 1.9	↑ Flatulence (15 g/d), breath H_2_ excretion (15 g/d)↔ Stool frequency, abdominal pain or cramps, stool forms or bloating
(20)	R, DB, SC, PC, P	Maltodextrin	Inulin (Beneo-Orafti HSI)	NA	8	4	NA	36 (18)	Healthy adults with GI symptoms age 20–70 y	↔ Transit time, stool frequency, stool consistency↓ Gas retention
(72)	R, DB, PC, CF, DR	Saccharose	Fibrulose 97 Fibruline instant (FI)Fibruline XL (FXL)	F97: 2–20FI: 2–60 (10)FXL: 2–60 (>20)	0, 5, 10, 20	10	2	84	Healthy adults age 30.1 ± 8.6 y, BMI 25.1 ± 3.1	↑ Flatulence, gas, bloating↔ Stool frequency, stool consistency, gastrointestinal rumbling
(27)	R, DB, PC, CF	No ITF	Inulin (Beneo HP, DKSH/Orafti)	≥23	10	1	2	21	Adults with overweight/obesity age 60 y, BMI 25–40	↑ Breath H_2_, stomach discomfort, nausea, flatulence, heartburn, and frequency↔ Bloating, belching
(29)	R, P	Low nonstarch polysaccharide diet	Chicory inulin (Agro-industries, Recherches et Developpements Society)	NA	2 wk @ progressive intake, 12 d @ 50	3.7	NA	9	Healthy adult men age 21.5 + 2.5 y, BMI <25	↑ Defecation, wet stool weight↔ Dry fecal weight
(32)	R, DB, PC, CF	Maltodextrin	Inulin–oligofructose (Synergy 1, Beneo-Orafti)	Oligofructose: 2–8 (4)Inulin: 10–60 (24)	15	3	2	30	Healthy adults age 28.1 ± 5.1 y, BMI 24.2 ± 3.0	↑ Gas, bloating, cramping, indigestion↔ Daily bowel movements
(33)	R, DB, PC, CF	Maltodextrin	VLCI, Bayer Cropscience AG) + Inulin (Beneo HP, Beneo-Orafti)	VLCI: 50–103HP: ≥23	10	3	3	31	Healthy adults age 25 y, BMI 20–30	↑ Bloating, gas production↔ Daily bowel movements, stool consistency, abdominal pain, flatulence
(73)	R, DB, P, PC	Maltodextrin	scFOS	NA	10	4	NA	244	Healthy adults age 50 y	↑ Flatulence, sense of “well-being,” stool frequency and regularity (in those with complaints of diarrhea), stool hardness (in those with constipation)↔ Post-study: bloating, flatulence, constipation, greater regularity, reduced IBS symptoms↓ Stool size (in those with constipation)
(34)	R, SB, PC, CF	No ITF	Inulin (Inulin HP, Beneo-Orafti)	≥23	22.4	1	1	13	Healthy adults age 23 ± 4 y, BMI 22.1 ± 1.6	↑ H_2_, flatulence
(35)	R, SB, PC, P	Cellulose+Maltodextrin	Oligofructose (Beneo P95, Beneo-Orafti)	2–7	30	6	NA	22 (12)	Healthy adults with overweight and obesity, 20–49 y, BMI 30.33	↑ Breath H_2_, flatulence, bloating, abdominal pain↔ Symptoms of diarrhea, stomach discomfort
(74)	R, DB, CF	Sucrose	Inulin (Raftiline ST, Beneo-Orafti)	2–65 (10)	18	6	6	21	Adults with mild-to-moderate hypercholesterolemia (LDL 3.36–5.17), age 60.4 y, BMI 27.99	↑ Flatulence, cramping, bloating, frequency, loose stool
(75)	R, PC, DB, CF	Molasses	Yacon (scFOS)	2–10	20	2	2	16	Healthy adults age 29.3 ± 4.9 y	↑ Stool frequency and softer stool consistency (trending)↔ Left colon or sigmoid rectum transit time, mean number of days with bloating↓ Colonic transit time
(76)	R, DB, CF	Maltodextrin	scFOS (Raftilose P95, Orafti)	<10	10	5	NA	20	Healthy adults age 28 y, BMI 22.5	↑ Belching, bloating (males), abdominal pain (males), urgency (rectal barostat)↔ Women only: bloating, frequency, borborygmi, pain, belching, nausea
(40)	SGD	Habitual diet	ITF-rich vegetables (artichoke, garlic, salsify, shallot, leek, scorzonera, onion, celery root)	NA	∼15	2	3	26	Healthy adults age 21.84 ± 0.39 y, BMI 22.29 ± 0.32	↑ Flatulence↔ Transit time, stool frequency, stool consistency, bloating, rumbling, cramp, and nausea↓ Intestinal discomfort, bloating (during fasting after intervention)
(77)	R, DB, PC, CF	Placebo	Inulin (BIOAGAVE, Ingredion)	25–34	0, 5, 7.5	3	1	29	Healthy adults age 27 ± 4.1 y, BMI 24.4 ± 2.26	↑ Bloating, flatulence, rumbling, bowel movement, breath H_2_, softer stools, abdominal pain (daily only), rumbling (daily only)↔ Weekly abdominal, nausea, ease of stool passage↓ Stool dry matter
(42)	R, DB, PC, CF	Maltodextrin	scFOS	NA	16	1.4	3	20	Adults with IBS (diarrhea-predominant or mixed), age 34.6 y, BMI 26.9	↑ Severity and frequency of abdominal pain, severity of abdominal distention, VAS IBS severity, nausea/vomiting, headache, belching, flatulence
(43)	R, DB, PC, P	Cereal mixture	Artichoke inulin (Liven GmbH)Chicory inulin (Fibruline Instant, Cosucra)	5–12	1 wk @ 7.72 wk @ 15.4	3	NA	45 (15)	Healthy adults age 23.5 ± 2.3 y, BMI 22.4 ± 2.7 (F) 23.4 ± 1.4 (M)	↑ Stool frequency, flatulence, stool consistency
(45)	CF	No inulin	Inulin (Fibruline, Cosucra)	2–50 (9)	22–34 (25% of energy from CHO)	9.14	4.9	8	Healthy adults age 26–53 y, BMI 22.8 ± 4.4 (F) 25.8 ± 1.6 (M)	↑ Flatulence, bloating↔ Diarrhea, nausea, stool consistency
(46)	P	No ITF	Inulin–oligofructose (Raftiline HP + Raftilose P95, Beneo-Orafti)	HP: 5–60 (10)P95: 2–7	15	2	NA	29 (14)	Adults undergoing colonoscopy age 35–72 y	↑ Flatulence, bloating, laxation
(47)	R, DB, PC, P	Maltodextrin	Inulin–oligofructose (Synergy 1, Beneo-Orafti)	Oligofructose: 2–8 (4)Inulin: 10–60 (24)	8	4	NA	43 (22)	Healthy adults age 45–63 y, BMI 25.36	↑ Flatulence, bowel movements↔ Constipation, stool consistency
(78)	R, DB, PC,P, MC	Glucose	scFOS (Idolax, Orafti)	NA	2 wk @ 10, 10 wk @ 20	12	NA	96 (50)	Adults with IBS age 45.1 ± 13.1 y	↑ Flatulence, defecation frequency↔ Weekly defecation frequency, IBS symptoms
(79)	R, DB, PC, P	Maltodextrin	Oligofructose (Raftilose P95)	2–7	21	12	NA	39 (21)	Adults with overweight/obesity age 40.4 y, BMI 30.1	↑ Flatulence↔ Diarrhea, nausea
(80)	R, DB, CF	No ITF	Inulin (Raftilin LS, Orafti)	11–12	14	4	0	72	Healthy women age 20–36 y, BMI 21.9 ± 2.6	↑ Flatulence, rumbling, cramps, bloating
(81)	R, TB, PC, P	Maltodextrin	Oligofructose (Fructalose L92, Sensus)	2–10	1 wk @ 8, 11 wk @ 16	12	NA	55 (29)	Adults with overweight/obesity age 41 ± 12 y, BMI 29.4 ± 2.6	↑ Flatulence (compared with control) and perceived bloating↔ Looser stools, regurgitation, nausea
(50)	R, DB, DR, PC, CF	Placebo	ITF	NA	0, 3, 7	4	4	48	Healthy adults age 30.85 y, BMI 23.98	↑ Bloating, abdominal distention
(51)	R, DB, PC, P	Placebo	Inulin (4 g) + oligofructose (12 g)	NA	2 wk @ 8, 10 wk @ 16	12	NA	96 (26)	Adults with overweight/obesity, 39.8 y, BMI 31.5	↑ Bloating, flatulence, rumbling↔ Looser stools, regurgitation and nausea
(82)	DB, CF	Sucrose	Inulin-rich soluble chicory extract	3–60	5, 7.8	4	NA	53	Healthy adults age 18–67 y, BMI 22.05	↑ Abdominal discomfort (7.8 g/d dosage)↔ Flatulence, abdominal pain, stool consistency and frequency
(83)	R, DB, PC, CF	No ITF	Inulin (Raftiline HP-Gel, Orafti)	>23	11	5	8	20	Healthy adult men, 18.8 ± 0.7 y, BMI 22.8 ± 2.3	↑ Gastric emptying rate↔ Flatulence, meteorism, bowel habit
(84)	R, DB, PC, CF	No ITF	Inulin (Raftiline HP-Gel, Orafti)	>23	11	5	8	20	Healthy adult men age 18.8 ± 0.7 y, BMI 22.8 ± 2.3	↓ Lactulose, zonulin
(53)	R, DB, PC, CF	Maltodextrin	scFOS (Raftilose P95)	2–7	25–30	2	2	12	Healthy adults age 21.4 ± 2 y, BMI 20.75	↑ Stool frequency, flatulence↓ Fecal water cytotoxicity
(54)	R, DB, PC, CF	Corn syrup	Inulin (Frutafit, Sensus)	9 (2–60)	20	3	0	12	Healthy adult men age 27–49 y, BMI <32	↑ Flatulence↔ Stool weight, transit time, stool frequency, and stool consistency
(55)	SC, R, DB, PC, P	Maltodextrin	Oligofructose (Orafti P95, Beneo-Orafti)	2–8	14	1	NA	37 (19)	Healthy adults age 25 y, BMI 23	↑ Breath H_2_, total colonic volume (ascending, transverse, distal)↔ Transit scores
(56)	R, DB, PC, P	Sucrose	scFOS (NutraFlora, GTC Nutrition)	3–5	3	4	NA	62 (15)	Healthy adults age 25.3 y	↔ Stools, fecal scores
(59)	R, DB, PC, CF	Sucrose	scFOS (Raftilose P95)	2–7	20	2	2	34	Healthy adult men age 27.7 ± 1.7 y, BMI 23.2 ± 0.5	↑ Flatulence, bloating, fecal wet weight, fecal lactic acid excretion, fecal mucin excretion↔ Fecal water cytotoxicity
(61)	R, DB, CF, LS	Basal diet	InulinscFOS	NA	15	3	0	12	Healthy adult men age 23 ± 3 y, BMI 23	↑ Breath H_2_ excretion (scFOS)
(62)	R, DB, DR, PC, CF	Sucrose	Oligofructose (Fructalose L92, Sensus)	2–10	1 wk @ 15, 1 wk @ 30	2	2	19	Healthy adults age 46.9 y, BMI 24.4	↓ Fecal water cytotoxicity

1 CF, crossover feeding; CHO, carbohydrate; DB, double-blinded; DP, degree of polymerization; DR, dose ranging; FI, Fibruline Instant; FOS, fructooligosaccharide; FXL, Fibruline XL; GI, gastrointestinal; H_2_, hydrogen; IBS, irritable bowel syndrome; ITF, inulin-type fructans; LS, Latin square; MC, multiple crossover; MCF, multiple crossover feeding; NA, not applicable; OLS, orthogonal Latin-square; P, parallel; PC, placebo-controlled; Pr, prospective; R, randomized; Ref, reference; SB, single-blinded; SC, single-center; scFOS, short-chain fructooligosaccharides; SGD, single-group design; TB, triple blind; VAS, visual analogue scale; VLCI, very long chain inulin.

One study measured intestinal permeability (84) and reported lower serum zonulin values in the inulin group (11 g/d) compared with both baseline and a low-dose (1.4 g/d inulin) control group. In addition to lower serum zonulin values in the inulin group (84), the urinary lactulose–mannitol excretion ratio was lower in the inulin group than in the control group and at baseline (84). Three studies measured fecal cytotoxicity, with 2 studies testing the same scFOS variant; however, whereas 1 study reported a statistically significant decrease in fecal water cytotoxicity (25–30 g/d scFOS) (53), the other reported no change (20 g/d scFOS) (59). The third study included oligofructose (15–30 g/d) in the intervention and reported a reduction in fecal water cytotoxicity compared with placebo (62).

Transit time measured by a hydrogen breath test (40) and plastic radio-opaque pellets (20, 54) demonstrated no change compared with their respective controls (20, 40, 54). Only 1 study reported a statistically significant reduction in transit time compared with the placebo group assessed by a radio-opaque marker technique (75). In this study, 6.4 g/d of scFOS syrup accelerated colonic transit time compared with the placebo group (75). The other studies testing inulin demonstrated no change compared with control or placebo (20, 40, 54). Qualitative assessments were used to measure stool frequency and consistency. For stool consistency, a scale (hard, formed, or soft), like the Bristol stool scale, was completed by participants. Stool frequency was measured in 20 ITF studies and was increased in 11 studies (27, 29, 34, 43, 47, 53, 73–75, 77, 78) but not in 9 other studies (18, 20, 32, 33, 40, 54, 72, 76, 82). Further, there was no change in stool consistency in 9 studies (18, 20, 33, 40, 47, 51, 54, 72, 81), though stool consistency became harder in 1 study (73) and softer in 4 studies (43, 74, 75, 77).

The most commonly reported measures of tolerance were flatulence, breath H_2_ gas, stool frequency, stool consistency, bloating, abdominal pain, and transit times. Assessments of flatulence, bloating, and abdominal pain were measured using a self-reported 4-point Likert scale (none, mild, moderate, or severe) or a visual analogue scale (VAS) of 100 mm (0 mm meant “no symptoms” and 100 mm corresponded to “unbearable symptoms”) (18, 27, 32–35, 40, 42, 43, 45–47, 50, 53, 54, 59, 72–74, 76–84). Most studies (19/37) measuring flatulence reported statistically significant increases in severity, indiscriminate of the type of ITF consumed (18, 27, 34, 35, 40, 43, 45–47, 50, 53, 54, 59, 72–74, 78–81). Only 3 reported no change in VAS scores for flatulence with consumption of 8.1 g/d of inulin (71) and 11 g/d of inulin (66, 67). Severity scores of abdominal pain were correlated with the ITF dose, with higher dosages resulting in higher reported severity scores (77). Participants in 5 studies reported an increase in severity scores for abdominal pain after consuming an ITF (27, 35, 42, 76, 77, 82), 2 studies reported no changes in scores (18, 33), and 1 study reported a 52% reduction in abdominal sensation after consuming inulin (8 g/d) (20). There were statistically significant increases in severity scores of bloating in 12 studies (32, 33, 35, 45, 50, 51, 59, 72, 74, 76, 77, 80) and no change in 3 studies (18, 27, 40). Similar to flatulence, breath H_2_ was indiscriminate to the type of ITF given to subjects and was higher compared with control (18, 20, 27, 32–35, 55, 61, 72, 77). However, symptoms tended to alleviate with time, indicating an adaptation to ITF intervention.

#### Glucose homeostasis

ITF have the potential to influence glucose homeostasis by both direct and indirect mechanisms. ITF are fibers and thus directly reduce carbohydrate availability and slow gastric emptying during digestion (64). Additionally, fermentation of ITF by the intestinal microbiota results in the production of SCFAs, which stimulate the release of incretin hormones, including glucagon-like peptide-1 (GLP-1) and peptide YY (PYY) (64, 85). Effects of ITF on incretin hormones, glucose and insulin (fasting and postprandial), insulin resistance or sensitivity, and hemoglobin A1c (HbA1c) are reported in **Table 3**(27, 30, 34–36, 38, 57, 61, 79, 86–91). Four studies included both SCFA and glucose homeostasis outcomes (30, 34, 35, 61), of which 3 included serum SCFAs (30, 34, 35). As mentioned above, most studies reported no effect of ITF on fasting or circulating SCFAs (Table 1). However, Daud et al. reported that 30 g/d of oligofructose (DP 2–7) for 6 wk in adults who were overweight and obese was associated with higher postprandial serum acetate concentrations compared with placebo (cellulose) as well as increased fasting serum propionate and butyrate concentrations compared with baseline (35). The changes in serum SCFA concentrations were accompanied by higher postprandial PYY concentrations, though fasting PYY and both fasting and postprandial GLP-1 did not differ between treatments (35). Cani et al. also reported higher postprandial PYY and GLP-1 following consumption of an inulin–oligofructose blend (16 g/d) compared with control, though fasting levels of both hormones as well as fasting and postprandial levels of glucose-dependent insulinotropic peptide (GIP) were unchanged and SCFAs were not measured (86). Similarly, Parnell et al. reported higher postprandial PYY following oligofructose consumption (21 g/d) compared with placebo, but no changes in GLP-1 or GIP were observed (79). Chambers et al. and Byrne et al. reported no effect of inulin (20 or 10 g/d, respectively) on SCFAs or incretin hormones, GLP-1 or PYY (27, 30).

**TABLE 3:** ITF effects on glucose homeostasis in healthy adults^1^

Ref.	Study design	Comparator	ITF type (trade name, manufacturer)	DP, range (mean)	ITF dosage, g/d	Intervention duration, wk	Washout duration, wk	N (*n*in ITF group)	Study population characteristics	Results
(27)	R, DB, PC, CF	No ITF	Inulin (Beneo HP, DKSH/Orafti)	≥ 23	10	1	2	21	Adults with overweight/obesity, 60 y, BMI 25–40	↔ Fasting or postprandial glucose, insulin, GLP-1, PYY
(86)	R, DB, PC, P	Dextrin maltose	Inulin–oligofructose (Synergy 1, Beneo-Orafti)	Oligofructose: 2–8 (4)Inulin: 10–60 (24)	16	2	NA	10 (5)	Healthy adults age 21–38 y, BMI 1.6 ± 0.99	↑ Postprandial GLP-1, PYY↓ Postprandial glucose↔ Fasting glucose, GLP-1, PYY, fasting or postprandial insulin, GIP, prancreatic polypeptide
(30)	R, DB, PC, CF	Cellulose	Inulin (Inulin HP, Beneo-Orafti)	≥23	20	6	≥3 (mean 6.3)	12	Nondiabetic adults with overweight/obesity, age 60 ± 1 y, BMI 29.8 ± 0.9	↓ Fasting insulin, insulin resistance (↓ HOMA2-IR, ↑ Matsuda)↔ Postprandial insulin, fasting or postprandial glucose, GLP-1, PYY, fecal or serum SCFAs
(34)	R, SB, PC, CF	No ITF	Inulin (Inulin HP, Beneo-Orafti)	≥23	22.4	1	1	13	Healthy adults age 23 ± 4 y, BMI 22.1 ± 1.6	↔ Serum SCFAs, insulin sensitivity (HOMA), postprandial glucose/insulin
(35)	R, SB, PC, P	Cellulose + Maltodextrin	Oligofructose (Beneo P95, Beneo-Orafti)	2–7	30	6	NA	22 (12)	Healthy adults with overweight and obesity, 20–49 y, BMI 30.33	↑ Postprandial PYY, serum acetate (vs. placebo), fasting serum propionate and butyrate (vs. baseline)↔ Fasting PYY, fasting or postprandial GLP-1, glucose, insulin
(36)	R, DB, PC, P	Maltodextrin	Inulin–oligofructose (Synergy 1, Beneo-Orafti)	Oligofructose: 2–8 (4)Inulin: 10–60 (24)	16	12	NA	30 (15)	Adult women with obesity, 47.5 y, BMI 35.85	↓ Post-OGTT glucose↔ HbA1c, fasting glucose, fasting or post-OGTT insulin, HOMA
(38)	R, DB, PC, CF	Maltodextrin + aspartame	scFOS (Actilight P950, Beghin Meiji)	2–4	10.6	8	4	30	Adults with mild hypercholesterolemia (TC 5.17–7.76 mmol/L and TG < 3.45 mmol/L), 45.5 ± 9.9 y, BMI 26.6 ± 2.2	↓ Postprandial insulin iAUC↔ Fasting glucose, insulin, acetate, postprandial glucose
(87)	R, DB, PC, CF	Cellulose	Inulin–oligofructose (Synergy 1, Orafti-Beneo)	Oligofructose: 2–8 (4)Inulin: 10–60 (24)	4 wk @ dose-escalation, 2 wk @ 30	6	4	34	Adults with prediabetes, 61.9 y, BMI 28.83	↑ Postprandial insulin iAUC↔ Fasting insulin, fasting and postprandial glucose, insulin resistance (HOMA-IR)↓ Fasting insulin, HOMA-IR in participants with impaired fasting glucose subtype
(88)	R, DB, PC, P	Maltodextrin	Inulin (Raftiline HP, Beneo-Orafti)	5–60 (10)	10	8	NA	54 (27)	Healthy middle-aged adults age 52.2 ± 9.5 y, BMI 26.4 ± 3.2	↓ Fasting insulin↔ Fasting glucose
(89)	R, DB, PC, CF	Sucrose	scFOS (Actilight 950P)	2–4	20	4	2	12	Healthy adult men, 24 ± 1 y, BMI 21 ± 0.5	↓ Basal hepatic glucose production↔ Fasting glucose/insulin, insulin sensitivity (euglycemic hyperinsulinemic clamp)
(79)	R, DB, PC, P	Maltodextrin	Oligofructose (Raftilose P95, Beneo-Orafti)	2–7	21	12	NA	39 (21)	Adults with overweight/obesity, 40.4 y, BMI 30.1	↑ Postprandial PYY↓ Postprandial glucose, insulin↔ GLP-1, GIP
(57)	R, DB, DR, PC, P	Maltodextrin	scFOS (Fossence, Tata Chemicals Limited)	3–5	0, 2.5, 5, 10	12.85	NA	80 (20)	Healthy adults age 23–44 y, BMI 24 ± 3.2	↓ Glucose
(90)	R, DB, PC, CF	Refined wheat	Inulin	NA	15	4	4	10	Adult men at higher risk of CVD	↔ Post-OGTT glucose/insulin
(61)	R, DB, CF, LS	Basal diet	InulinscFOS	NA	15	3	0	12	Healthy adult men, 23+3 y, BMI 23	↑ Fecal acetate and valerate (inulin)↔ Post-OGTT glucose/insulin, other SCFAs
(91)	R, DB, PC, CF	Cellulose	Inulin (Fibruline DS2, Georg Breuer GmbH)	NA	30	1	1	16	Healthy adults age 40.5 ± 4.2, BMI 23.1 ± 1.0	↔ Fasting glucose, insulin

1 CF, crossover feeding; CHO, carbohydrate; CVD, cardiovascular disease; DR, dose ranging; DB, double-blinded; DP, degree of polymerization; GIP, glucose-dependent insulinotropic peptide; gLP-1, Glucagon-like peptide-1; HbA1c, hemoglobin A1c; IR, insulin resistance; ITF, inulin-type fructans; LS, Latin square; MCF, multiple crossover feeding; NA, not applicable; OGTT, oral glucose tolerance test; OLS, orthogonal Latin-square; P, parallel; PC, placebo-controlled; Pr, prospective; PYY, peptide YY; R, randomized; SB, single-blinded; SC, single-center; scFOS, short-chain fructooligosaccharides; SGD, single group-design; TB, triple blind; TC, total cholesterol; TG, triglycerides.

ITF had no effect on fasting glucose (27, 30, 35, 36, 38, 61, 86–88, 90–92), although Cani et al. and Parnell et al. reported a decrease in or lower concentrations of postprandial glucose after consumption of a standardized meal following the ITF treatment (79, 86). Dewulf et al. observed a decrease in glucose following an oral glucose tolerance test (OGTT) after 3 mo of consuming an inulin–oligofructose blend (16 g/d), but did not detect any changes in HbA1c (36). Fasting insulin was not affected by ITF intake in the majority of studies (27, 35, 36, 38, 61, 89–91), although 2 studies reported a decrease in or lower concentrations of fasting insulin following consumption of long-chain inulin (30, 88). In a study by Guess et al. in adults with prediabetes (87), there was an increase in postprandial insulin but no effect of an inulin–oligofructose blend (30 g/d for 4 wk) on fasting insulin in the full cohort (87). However, when divided into prediabetic subtypes, fasting insulin was decreased in participants with an impaired fasting glucose prediabetic subtype (87). Although most studies observed no effect of ITF on postprandial or post-OGTT insulin (27, 30, 34–36, 61, 86, 90), 2 studies reported a decrease in or lower concentration of postprandial insulin (38, 79).

Whereas absolute concentrations of insulin may be used to estimate insulin sensitivity, the gold standard is a hyperinsulinemic euglycemic glucose clamp (93). Only 1 study used this method to measure insulin sensitivity, reporting no effect of scFOS (DP 2–4, 20 g/d for 4 wk) (89). The most common method used to estimate insulin sensitivity was the homeostatic model assessment (HOMA) (30, 34, 36, 87). Similar to reports for fasting insulin, Guess et al. reported no change in HOMA-IR in the full cohort but observed a decrease following intake of an inulin–oligofructose blend in participants with the impaired fasting glucose prediabetic subtype (87). Chambers et al. also reported a lower HOMA-IR and improved Matsuda insulin sensitivity index compared with the control (30). The remaining 2 studies reported no effect of ITF consumption on insulin sensitivity as measured by HOMA (34, 36).

Evidence for the role of the intestinal microbiota in mediating these effects is unclear. Tandon et al. reported no correlations between taxa abundances and blood glucose concentrations (57). Dewulf et al. reported an inverse correlation between change in *Bifidobacterium* abundance—which was increased by ITF—and changes in HbA1c, whereas the increase in *Clostridium* cluster IV by ITF was inversely correlated with changes in both HOMA and fasting glucose and insulin (36). In contrast, members of the genera *Bacteroides* and *Propionibacterium*, which were decreased by ITF, were positively correlated with changes in glucose homeostasis (36).

#### Cardiovascular health

ITF consumption may also exert a beneficial effect on cardiovascular health (8, 9). Markers of cardiovascular health that were measured included blood lipid levels, blood pressure, and arterial stiffness (**Table 4**) (21, 26, 27, 38, 45, 57, 61, 74, 78–80, 88–91, 94, 95). There is considerable support for the lipid-lowering effects of ITF in animal models, particularly on reducing triglyceride concentrations (8, 9). Tripkovic et al. investigated the effects of inulin (15 g/d) on markers of CVD, including blood pressure and arterial stiffness, in overweight men (90). No effects were reported on blood pressure, but a decrease in arterial stiffness was observed following inulin intake, though there were no differences between groups (90). Almost all studies only measured fasting lipid profiles, with just 1 study reporting postprandial triglyceride and free fatty acid concentrations (38). Three studies reported a decrease in or lower concentration of fasting serum and plasma triglycerides following ITF intake compared with baseline and/or placebo (26, 88, 95). However, 12 studies reported no effect on fasting triglycerides (27, 38, 45, 57, 61, 74, 80, 89–91, 94) or postprandial triglycerides (38). Two studies reported no changes in serum lipids or lipoproteins but did not specify which lipid outcomes were measured or provide values (78, 79). Total cholesterol was decreased or lower in 2 studies (26, 74) but was not affected by ITF intake in 8 studies (27, 38, 45, 61, 80, 88–90). One study reported a trend toward increased fecal cholesterol excretion following scFOS consumption (12.5 g/d), though blood lipids were not assessed (21). Another study reported decreased concentrations of fasting plasma total cholesterol and LDL cholesterol and increased concentrations of HDL cholesterol after consuming an inulin–oligofructose blend (10 g/d) compared with baseline, but these changes were not statistically different from those observed in the placebo group (94). One of the studies reporting lower serum total cholesterol following ITF consumption also reported a lower concentration of LDL cholesterol in adults with hypercholesterolemia consuming 18 g/d inulin (74). Conversely, HDL cholesterol was higher after ITF consumption (11 g/d) in 1 study (95), which led to a lower total cholesterol/HDL cholesterol ratio (95). However, no statistically significant changes were observed in the LDL/HDL cholesterol ratio (27, 45, 74, 94). In addition, 6 studies reported no differences in LDL cholesterol following ITF consumption (26, 27, 38, 45, 61, 80, 88), although 9 studies reported no effect on HDL cholesterol (26, 27, 38, 45, 61, 80, 88–90, 96). VLDL (38) and free fatty acid (27, 38, 90) concentrations were also reported to have no effect of ITF intake. Plasma concentrations of odd-chain fatty acids, such as pentadecanoic acid and heptadecanoic acid, were increased following 1 wk of inulin intake (30 g/d) (91). Odd-chain fatty acids are inversely correlated with risk of type 2 diabetes (91). Therefore, this increase following inulin consumption suggests that odd-chain fatty acids may be targets of ITF on the lipid profile and health.

**TABLE 4:** ITF effects on cardiovascular health in healthy adults^1^

Ref.	Study design	Comparator	ITF type (trade name, manufacturer)	DP, range (mean)	ITF dosage, g/d	Intervention duration, wk	Washout duration, wk	N (*n*in ITF group)	Study population characteristics	Results
(21)	R, DB, PC, P	Saccharose	scFOS (Actilight, Eridania-Beghin Say)	2–4	12.5	1.7	1.7	20	Healthy adults age 22–39 y	↑ Fecal cholesterol (trend)↔ Fecal bile acids, neutral sterols (coprostanol, epicoprostanol, cholestanol)
(26)	PC, CF, SB	Cereal (rice)	Inulin (Fibruline Instant, Cosucra)	2–60 (10)	9	4	4	12	Healthy adult men, 23.3 ± 0.5 y, BMI 25.7 ± 1.2	↔ Fasting serum LDL-C, HDL-C, total/HDL-C ratio, fecal bile acids↓ Fasting serum TG, TC*Bif* and total anaerobes ∼ TG (–)Bifidobacterium ∼ HDL-C (+), total/HDL-C (–)Total facultative anaerobes ∼ TC (–)Butyrate ∼ total and LDL-C (+) Propionate ∼ HDL-C (–)Litocholic acid and secondary bile acids ∼ TC and TG (+), total/HDL-C ratio (–)
(27)	R, DB, PC, CF	No ITF	Inulin (Beneo HP, DKSH/Orafti)	≥23	10	1	2	21	Adults with overweight/obesity, age 18–65 y, BMI 25–40	↔ Fasting TG, TC, HDL-C, LDL-C, LDL-C/HDL-C, total/HDL-C, fasting or postprandial non-esterified fatty acids
(74)	R, DB, CF	Sucrose	Inulin (Raftiline ST, Beneo-Orafti)	2–65 (10)	18	6	6	21	Adults with mild-to-moderate hypercholesterolemia (LDL 3.36–5.17), 60.4 y, BMI 27.99	↓ Fasting serum TC, LDL-C↔ Fasting serum TG, HDL-C, LDL-C/HDL-C ratio
(94)	R, DB, PC, P	Maltodextrin	Inulin–oligofructose (Synergy 1, Beneo-Orafti)	Oligofructose: 2–8 (4)Inulin: 10–60 (24)	10	24	NA	17 (9)	Healthy adults age 31.5 y	↑ Plasma HDL-C (trending)↔ Plasma TG, hepatic lipogenesis, cholesterol synthesis, mononuclear cell mRNA↓ Plasma TC (trending), LDL-C/HDL-C (trending), LDL-C (trending)
(38)	R, DB, PC, CF	Maltodextrin + aspartame	scFOS (Actilight P950, Beghin Meiji)	2–4	10.6	8	4	30	Adults with mild hypercholesterolemia (TC 5.17–7.76 mmol/L, TG < 3.45 mmol/L), age 45.5 ± 9.9 y, BMI 26.6 ± 2.2	↑ Fasting plasma Lp(a)↔ Fasting plasma cholesterol, VLDL, LDL-C, HDL-C, apoA-I; fasting and postprandial plasma TG and free fatty acids
(88)	R, DB, PC, P	Maltodextrin	Inulin (Raftiline HP, Beneo-Orafti)	5–60 (10)	10	8	NA	54 (27)	Healthy middle-aged adults age 52.2 ± 9.5 y, BMI 26.4 ± 3.2	↓ Fasting plasma TG (higher baseline TG associated with greater decrease)↔ Fasting plasma TC, HDL-C, LDL-C, apoB, apoA-I
(45)	CF	No inulin	Inulin (Fibruline, Cosucra)	2–50 (9)	22–34 (25% of energy from CHO)	9.14	4.9	8	Healthy adults age 26–53 y, BMI 22.8 ± 4.4 (F) 25.8 ± 1.6 (M)	↔ Fasting serum TG, TC, HDL-C, LDL-C, LDL-C/HDL-C ratio
(89)	R, DB, PC, CF	Sucrose	scFOS (Actilight 950P)	2–4	20	4	2	12	Healthy adult men, 24 ± 1 y, BMI 21 ± 0.5	↔ Fasting serum TG, TC, HDL-C, apoA-I, apoB, Lp(a)
(78)	R, DB, PC, P, MC	Glucose	scFOS (Idolax, Orafti)	NA	2 wk @ 1010 wk @ 20	12	NA	96 (50)	Adults with IBS age 45.1 ± 13.1 y	↔ Serum lipids and lipoproteins
(79)	R, DB, PC, P	Maltodextrin	Oligofructose (Raftilose P95, Beneo-Orafti)	2–7	21	12	NA	39 (21)	Adults with overweight/obesity, 40.4 y, BMI 30.1	↔ Serum lipids
(80)	R, DB, CF	No ITF	Inulin (Raftilin LS, Orafti)	11–12	14	4	0	72	Healthy women, 20–36 y, BMI 21.9 ± 2.6	↔ Fasting plasma TG, TC, HDL-C, LDL-C
(95)	R, DB, PC, CF	Refined wheat	Inulin (Raftiline HP-Gel, Orafti)	>23	11	5	8	22	Healthy adult men age 18.8 ± 0.7 y, BMI 22.8 ± 2.3	↑ Fasting serum HDL-C↓ Fasting serum TG, total/HDL-C ratio, plasma Lp(a)
(57)	R, DB, DR, PC, P	Maltodextrin	scFOS (Fossence, Tata Chemicals Limited)	3–5	0, 2.5, 5, 10	12.85	NA	80 (20)	Healthy adults age 23–44 y, BMI 24 ± 3.2	↔ Triglycerides
(90)	R, DB, PC, CF	Refined wheat	Inulin	NA	15	4	4	10	Adult men at higher risk of CVD, 39.9 ± 9 y, BMI 30.2 ± 3	↔ Fasting plasma TG, TC, HDL-C, non-esterified fatty acids↔ Blood pressure, arterial stiffness
(61)	R, DB, CF, LS	Basal diet	InulinscFOS	NA	15	3	0	12	Healthy adult men, 23 ± 3 y, BMI 23	↓ Fecal deoxycholic acid↔ Fasting serum TG, TC, HDL-C, LDL-C, apoA-I, apoB, other fecal bile acids
(91)	R, DB, PC, CF	Cellulose	Inulin (Fibruline DS2, Georg Breuer GmbH)	NA	30	1	1	16	Healthy adults age 40.5 ± 4.2 y, BMI 23.1 ± 1.0	↑ Pentadecanoic acid, heptadecanoic acid↔ Fasting serum TG

1 C, cholesterol; CF, crossover feeding; CHO, carbohydrate; CVD, cardiovascular disease; DR, dose ranging; DB, double-blinded; DP, degree of polymerization; IBS, irritable bowel syndrome; ITF, iInulin-type fructans; Lp(a), lipoprotein (a); LS, Latin square; MCF, multiple crossover feeding; NA, not applicable; OLS, orthogonal Latin square; P, parallel; PC, placebo-controlled; Pr, prospective; R, randomized; SB, single-blinded; SC, single-center; scFOS, short-chain fructooligosaccharides; SGD, single-group design; TB, triple blind; TC, total cholesterol; TG, triglycerides.

Variants or components of lipoproteins including lipoprotein(a) [Lp(a)], apoB, and apoA-I may also modulate disease risk (97–99). No effects of ITF consumption on serum or plasma apoB or apoA-I concentrations were reported (38, 61, 88, 89). However, contrasting effects on Lp(a) concentrations were observed (38, 95). Russo et al. reported lower concentrations of fasting serum Lp(a) following consumption of long-chain inulin (11 g/d) in healthy adult men (95), whereas Giacco et al. reported higher concentrations of fasting plasma Lp(a) following consumption of scFOS (10.6 g/d) in adults with mild hypercholesterolemia (38). The lack of consistent effects on Lp(a), apoB, and apoA-I suggest that ITF consumption does not modify lipoprotein composition.

Previous reviews have reported a greater effect of long-chain inulin than scFOS on lipid profiles in humans (8, 9). The current analysis supports this conclusion. Although results were mixed, studies reporting a decrease in triglycerides or total cholesterol all tested long-chain inulin (26, 74, 88, 95). However, only 1 study reporting lipid profile outcomes compared the effects of inulin and scFOS (15 g/d) and reported no effects of either on lipid profiles (61). Individuals’ intestinal microbiota or baseline metabolic indices may also impact the effect of ITF intake on lipid metabolism. For instance, Brighenti et al. observed several correlations between participants’ fecal microbiota composition and lipid profiles, including associations between *Bifidobacterium* and triglycerides (negative) as well as HDL cholesterol (positive), total anaerobes with triglycerides (negative), total facultative anaerobes with total cholesterol (negative), and secondary bile acids with total cholesterol and triglycerides (positive) (26). Additionally, Jackson et al. observed a positive correlation between baseline triglyceride concentration and decreases in triglycerides following inulin consumption (i.e., higher baseline triglycerides associated with a greater decrease) (88).

#### Mineral absorption and bone health

ITF may also impact mineral absorption (48). Animal studies consistently report a positive effect of ITF on mineral absorption, particularly calcium and magnesium, and bone mineral density (8, 9). However, caution is warranted in the interpretation and translation of findings from animal studies, as the mechanisms of mineral absorption may differ. For example, calcium absorption occurs primarily in the upper portion of the small intestine in humans compared with the large intestine in rats (100), which may cause differences in the effects of ITF due to differences in pH and SCFA concentration (101). In animals, the positive effects of ITF on mineral absorption have been shown to be inversely correlated with baseline mineral status or absorptive capacity, which typically declines with age (8, 9). In humans, the majority of studies on ITF and mineral absorption and bone health have been conducted in adolescents and postmenopausal women (8, 9). The current review focuses on the prebiotic effects of ITF in adults and therefore did not include the effects of ITF in adolescents.

When mineral absorption and bone health were the focus, studies most commonly investigated the effects of ITF on calcium absorption (57, 100, 102–106) (**Table 5**) (48, 57, 100, 102–108). Despite differences in populations (e.g., young adults, adult men, postmenopausal women), 4 studies reported an increase in intestinal calcium absorption and calcium balance (excretion – absorption) following ITF intake (57, 100, 102, 103). Two of these studies used stable isotopes measured in serum (100) or plasma (103) to measure calcium absorption, whereas 1 calculated absorption based on intake minus fecal concentrations (102). Based on isotopic tracing methods, the majority (70%) of this increase was associated with colonic phase absorption (100). However, 3 studies reported no effect of ITF consumption on serum calcium concentrations or calcium absorption (104–106). However, serum calcium is tightly regulated by parathyroid hormone (PTH), vitamin D, and serum calcium itself, which together dictate calcium transport in the gut, kidney, and bone (109). Therefore, effects on these regulatory factors and on bone mineral density provide further clarity regarding effects on bone health.

**TABLE 5:** ITF effects on mineral absorption and bone health in healthy adults^1^

Ref.	Study design	Comparator	ITF type (trade name, manufacturer)	DP, range (mean)	ITF dosage, g/d	Intervention duration, wk	Washout duration, wk	N (*n*in ITF group)	Study population characteristics	Results
(100)	SGD	No ITF	Inulin–oligofructose (Synergy 1, Beneo-Orafti)	Oligofructose: 2–8 (4)Inulin: 10–60 (24)	8	8	NA	13	Healthy young adults age 23.9 ± 2.1 y, BMI 22.2 ± 2.2	↑ Calcium absorption (serum isotopes)8/13 “responders” (≥3% ↑ calcium absorption)
(102)	R, P	Low nonstarch polysaccharide diet	Chicory inulin (Agro-industries, Recherches et Developpements Society)	NA	2 wk @ progressive intake, 12 d @ 40	3.7	NA	9	Healthy adult men, 21.5 ± 2.5 y, BMI < 25	↑ Calcium absorption/balance↔ Magnesium, iron, zinc absorption/balanceAbsorption (intake − feces)Balance (urinary excretion − absorption)
(103)	R, DB, PC, CF	Maltodextrin	Inulin–oligofructose (Synergy 1, Beneo-Orafti)	Oligofructose: 3–8 (4)Inulin: 10–65 (25)	10	6	6	15	Healthy postmenopausal women age 72.2 ± 6.4 y, BMI 25.2 ± 3.3	↑ Calcium and magnesium absorption (plasma isotopes), bone resorption (deoxypyridinoline), bone formation (osteocalcin)↔ Serum vitamin D, PTH∼2/3 “responders” (↑ calcium & magnesium absorption)Baseline lumbar spine BMD ∼ responders (–)Baseline bone turnover ∼ magnesium absorption response (+)
(48)	R, DB, PC, CF	Maltodextrin	Inulin (Fibruline Instant, Cosucra)	2–60 (10)	20	4	2	32	Adult women with low iron (plasma ferritin <25 μg/L), 18–40 y, BMI 21.5 ± 2.2	↔ Iron absorption (blood isotopes)
(104)	R, DB, PC, P	No ITF	scFOS	NA	2.5	96	NA	367 (152)	Healthy postmenopausal women age 59.7 y, BMI 28.0	↔ Serum vitamin D, calcium
(107)	R, DB, PC, P	Maltodextrin	scFOS (NutraFlora)	3–5	3.6	48	NA	300 (100)	Healthy postmenopausal women age 61 y, BMI 27.4	↓ Bone resorption (serum C-termal telopeptide), bone formation (serum osteocalcin)↔ Bone resorption (urinary deoxypyridinoline), serum vitamin D, calcium, plasma PTH↓ BMD loss
(108)	R, DB, PC, CF	Sucrose	scFOS (Actilight, Beghin Meiji)	2–4	4 d @ 5, 4.4 wk @ 10	5	≥3	11	Healthy postmenopausal women age 59 ± 6 y, BMI 25.1 ± 1.9	↑ Magnesium absorption/status
(105)	R, DB, PC, CF	Sucrose	scFOS (Actilight, Beghin Meiji)	2–4	10	5	≥3	12	Healthy postmenopausal women age 59.8 ± 6.2 y, BMI 25.2 ± 1.9	↑ Calcium absorption (trend in women with >6 y menopause)↔ Calcium absorption (fecal isotopes), calcium status (plasma/urine isotopes and totals), plasma vitamin D, PTH, bone resorption (urinary deoxypyridinoline), bone formation (plasma osteocalcin)↓ Plasma vitamin D (in women with 2–6 y menopause)
(57)	R, DB, DR, PC, P	Maltodextrin	scFOS (Fossence, Tata Chemicals Limited)	3–5	0, 2.5, 5, 10	12.85	NA	80 (20)	Healthy adults age 23–44 y, BMI 24 ± 3.2	↑ Serum calcium (2.5 g/d, trend for increase with 5 and 10 g/d doses)
(106)	R, DB, PC, CF	No ITF	InulinscFOS	Inulin: 2–60scFOS: 2–8	15	3	NA	12	Healthy adult men, 20–30 y	↔ Calcium, iron absorption (plasma/urine isotopes)

1 BMD, bone mineral density; CF, crossover feeding; DR, dose ranging; DB, double-blinded; DP, degree of polymerization; ITF, inulin-type fructans; MCF, multiple crossover feeding; NA, not applicable; P, parallel; PC, placebo-controlled; PF, plasma ferritin; PTH, parathyroid hormone; R, randomized; SB, single-blinded; SC, single-center; scFOS, short-chain fructooligosaccharides; SGD, single-group design; TB, triple blind.

There were no reported effects of ITF consumption on PTH or vitamin D concentrations in the studies included (103–105, 107), though subgroup analysis revealed that vitamin D was higher in women with early menopause (2–6 y) following scFOS intake (10 g/d) compared with placebo intake (105). Magnesium is also crucial for bone health (110). Effects on magnesium absorption were more positive. Two studies reported an increase in magnesium absorption and status following consumption of an inulin–oligofructose blend (10 g/d) or scFOS (10 g/d) (103, 108), though another study reported no effect of inulin (40 g/d) on magnesium absorption or balance (102). Despite positive effects on calcium and magnesium absorption, ITF effects on bone mineral density and markers of bone turnover have been mixed. Holloway et al. reported small increases in bone formation (indicated by serum osteocalcin concentrations) and bone resorption (indicated by urinary deoxypyridinoline concentrations) following 6 wk of consuming an inulin–oligofructose blend (10 g/d), though bone resorption showed a transient decrease after 3 wk of intake (103). Conversely, Slevin et al. reported higher concentrations of serum bone resorption marker C-telopeptide of type I collagen, though not urinary deoxypyridinoline or serum osteocalcin following scFOS consumption (3.6 g/d) for 48 wk compared with placebo (107). This resulted in a smaller decline in bone mineral density in the scFOS group (107). Tahiri et al. reported no effects of scFOS (10 g/d) consumption on urinary deoxypyridinoline or plasma osteocalcin concentrations after 5 wk (105).

In addition to effects on micronutrients related to bone health, a few studies have investigated the effects of ITF intake on iron and zinc absorption (48, 102, 106). However, no effects of ITF consumption were reported on the absorption of either of these micronutrients.

Factors that may influence the prebiotic effect of ITF on mineral absorption include age, baseline mineral status, and ITF chain length. For instance, responders—showing an increase in calcium and/or magnesium absorption—were reported to have a lower baseline lumbar spine bone mineral density and higher baseline bone turnover than nonresponders (103). Additionally, although no statistically significant effect on calcium absorption was observed in the full cohort, subgroup analysis revealed a trend for an increase in calcium absorption in women who had gone through menopause >6 y prior (105). Some evidence in rodents suggests that an inulin–oligofructose blend is more effective than oligofructose or inulin alone at stimulating calcium and magnesium absorption (14). Of the included clinical studies, those that demonstrated an increase in calcium absorption primarily utilized either an inulin–oligofructose blend or inulin alone (100, 102, 103), whereas those utilizing scFOS tended to show no effect on calcium absorption or bone (104–106). Only 1 study investigated correlations between mineral status, calcium, and the fecal microbiota, reporting no statistically significant associations (57).

#### Inflammation and immunity

Chronic inflammation contributes to a range of diseases, including inflammatory bowel disease, autoimmune diseases, cancer, and metabolic and neurogenetic disorders (111). The gastrointestinal microbiota contributes to the development and function of the immune system and modulates inflammatory processes locally and systemically via effects on epithelial cells and intestinal permeability, dendritic cells, and T and B immune cells (111, 112). These effects are mediated by microbial cell components, such as LPS, and metabolites, including SCFAs, and particularly butyrate (113). Fibers, particularly ITF, have been shown in animal models to reduce intestinal and systemic inflammation by increasing SCFA production and abundance of *Bifidobacterium* and *Lactobacillus* (9, 114). However, there is a lack of human intervention studies that test the effects demonstrated in experimental and preclinical models (9).

Results from the human clinical studies included in the current review indicate a small effect of ITF on certain components of the microbial–immune system interaction, though most measures of immune system function were not affected by ITF consumption (**Table 6**) (30, 32, 42, 47, 115). Changes in serum and fecal Ig concentrations can serve as markers for alterations in host adaptive immune function (32). Concentrations of salivary IgA and Ig isotypes (e.g., IgA, IgG, IgM) in blood were not affected by ITF intake (30, 32, 47). Parnell et al. reported a reduction in fasting plasma LPS concentrations following 12 wk of 21 g/d oligofructose consumption (115). However, Chambers et al. reported no change in serum LPS-binding protein concentrations after 6 wk of 20 g/d inulin (30) and Clarke et al. reported higher fasting serum LPS concentrations after 3 wk of 15 g/d of an inulin–oligofructose blend than those found after placebo consumption (32). Concentrations of proinflammatory C-reactive protein (30, 32) and the cytokine tumor-necrosis factor-α (32, 42, 47, 115) were not different following ITF intake. Other cytokines, including IL-2 (47), IL-4 (47), IL-6, (30, 42, 47, 115), IL-8 (30, 42), IL-10 (30, 47), IL-12 (30), IL-17A (30), monocyte chemoattractant protein-1 (115), and interferon-γ (47), were also largely unaffected by ITF consumption, though Clarke et al. reported higher concentrations of the T helper 2 cytokine IL-4 and lower concentrations of the regulatory cytokine IL-10 after consumption of an inulin–oligofructose blend compared with placebo (32). That study also reported a higher concentration of the proinflammatory cytokine granulocyte-macrophage colony stimulating factor (32). Conversely, Parnell et al. reported a decrease in the proinflammatory plasminogen activator inhibitor-1 (PAI-1) following oligofructose consumption (21 g/d) (116). PAI-1 is positively associated with inflammation and insulin resistance and may be regulated by proinflammatory cytokines (116). Cytokines produced by adipose tissue (i.e., adipokines) such as adiponectin and resistin have also been correlated with inflammatory markers, including PAI-1 (117), but were not changed following oligofructose intake (21 g/d) (115). Lastly, Clarke et al. reported higher numbers of cluster of differentiation 282+/toll-like receptor 2 + myeloid dendritic cells compared with placebo (32), but studies reported no other changes in immune cell populations or functionality, including T or B lymphocyte populations (30, 32, 47) or neutrophil and monocyte phagocytosis of *E. coli*, natural killer cell activity, and T cell activation and proliferation (30, 47).

**TABLE 6:** ITF effects on inflammation and immunity in healthy adults^1^

Ref.	Study design	Comparator	ITF type (trade name, manufacturer)	DP, range (mean)	ITF dosage, g/d	Intervention duration, wk	Washout duration, wk	N (*n*in ITF group)	Study population characteristics	Results
(30)	R, DB, PC, CF	Cellulose	Inulin (Inulin HP, Beneo-Orafti)	≥23	20	6	≥3 (mean 6.3)	12	Nondiabetic adults with overweight/obesity, 60 ± 1 y, BMI 29.8 ± 0.9	↔ Fasting serum IgA, IgG, IgM, CRP, LBP, IL-6, IL-8, IL-10, IL-12, IL-17A, T or B lymphocyte populations, T cell response
(32)	R, DB, PC, CF	Maltodextrin	Inulin–oligofructose (Synergy 1, Beneo-Orafti)	Oligofructose: 2–8 (4)Inulin: 10–60 (24)	15	3	2	30	Healthy adults age 28.1 ± 5.1 y, BMI 24.2 ± 3.0	↑ Fasting serum LPS, IL-4, GM-CSF, CD282+/TLR2 + mDC↔ Serum and fecal Ig (IgA, IgM, IgG), T or B lymphocyte populations, CRP, TNF-α↓ IL-10
(42)	R, DB, PC, CF	Maltodextrin	scFOS	NA	16	1.4	3	20	Adults with IBS (diarrhea-predominant or mixed), 34.6 y, BMI 26.9	↔ Serum IL-6, IL-8, TNF-α
(47)	R, DB, PC, P	Maltodextrin	Inulin–oligofructose (Synergy 1, Beneo-Orafti)	Oligofructose: 2–8 (4)Inulin: 10–60 (24)	8	4	NA	43 (22)	Healthy adults age 45–63 y, BMI 25.36	↔ Immune cell subsets, serum Ig (IgA, IgM, IgG), salivary IgA, neutrophil and monocyte phagocytosis of *E. coli*, natural killer cell activity, T cell activation/proliferation, cytokines (IL-2, IL-4, IL-6, IL-10, TNF-α, IFNγ)
(115)	R, DB, PC, P	Maltodextrin	Oligofructose (Raftilose P95, Beneo-Orafti)	2–7	21	12	NA	37 (20)	Adults with overweight/obesity, 40.4 y, BMI 30.1	↓ Fasting plasma PAI-1, LPS↔ Fasting plasma IL-6, TNF-α, MCP-1, adiponectin, resistin

1 CD, cluster of differentiation; CF, crossover feeding; CRP, C-reactive protein; DR, dose ranging; DB, double-blinded; DP, degree of polymerization; IBS, irritable bowel syndrome; GM-CSF, granulocyte-macrophage colony-stimulating factor; ITF, inulin-type fructans; MCF, multiple crossover feeding; MCP-1, monocyte chemoattractant protein-1; mDC, myeloid dendritic cells; NA, not applicable; P, parallel; PAI-1, plasminogen activator inhibitor 1; PC, placebo-controlled; R, randomized; SB, single-blinded; SC, single-center; scFOS, short-chain fructooligosaccharides; SGD, single-group design; TLR, Toll-like receptor.

There was no clear pattern in observed effects on immune system function based on ITF chain length. Regarding the role of the gastrointestinal microbiota, 2 of the included studies assessed correlations between immune markers and other outcomes (42, 47). However, Lomax et al. only assessed correlations between immune outcomes and BMI at baseline and did not assess correlations with microbial taxa (47). Hustoft et al. assessed correlations between proinflammatory cytokines, *Bifidobacterium*, Actinobacteria, *F. prausnitzii*, and SCFAs, but observed no statistically significant correlations (42).

#### Appetite and satiety

Appetite and satiety are components of the body's system of energy regulation and are important determinants of total energy intake. Appetite and satiety are controlled by a cascade of factors, including sensory and cognitive factors, stomach distension, and fluctuations in gut-derived hormones that communicate with the brain (e.g., cholecystokinin, ghrelin, GLP-1, PYY) (118). Additional gut peptides involved in satiety include neurotensin, somatostatin, and corticotropin-releasing factor, which regulate gastric emptying and gastrointestinal motility (83). These signals then lead to changes in subjective feelings of hunger or satiety and, potentially, changes in energy intake and body weight. Effects of ITF on measures of appetite and satiety are shown in **Table 7** (27, 30, 34, 35, 40, 51, 79, 81, 83, 86, 119–121). As discussed above, ITF can increase postprandial PYY, though effects on other gut-derived hormones such as GLP-1 and GIP were mixed (27, 30, 35, 79, 86, 119, 121). Both leptin and ghrelin were reported to be lower following 12 wk of oligofructose consumption (21 g/d) compared with a placebo in adults who were overweight and obese (79). The finding that both leptin (anorexigenic) and ghrelin (orexigenic) were lower following ITF compared with control makes it unclear how these changes may impact appetite and satiety. Russo et al. reported that inulin-enriched pasta (11 g/d inulin) increased neurotensin and somatostatin concentrations compared with baseline and a low-dose control pasta (1.4 g/d inulin) but had no effect on corticotropin-releasing factor (83). This study also reported that, compared with baseline and control, inulin-enriched pasta decreased gastric emptying rate, which was positively associated with plasma somatostatin concentration (83). The correlation between somatostatin and gastric emptying rate suggests that this hormone may be another mechanism by which inulin modulates satiety.

**TABLE 7:** ITF effects on appetite and satiety in healthy adults^1^

Ref.	Study design	Comparator	ITF type (trade name, manufacturer)	DP, range (mean)	ITF dosage, g/d	Intervention duration, wk	Washout duration, wk	N (*n*in ITF group)	Study population characteristics	Results
(27)	R, DB, PC, CF	No ITF	Inulin (Beneo HP, DKSH/Orafti)	≥23	10	1	2	21	Adults with overweight/obesity, 60 y, BMI 25–40	↔ Energy intake, composite appetite score, postprandial GLP-1, PYY
(119)	R, SB, PC, CF	Dextrin maltose	Oligofructose (Raftilose P95, Beneo-Orafti)	2–7	16	2	2	10	Healthy adults age 27.2 ± 1.6 y, BMI 22.3 ± 0.7	↑ Satiety @ breakfast and dinner (not at lunch)↓ Total energy intake; energy intake @ breakfast and dinner; hunger and prospective food consumption @ dinner
(85)	R, DB, PC, P	Dextrin maltose	Inulin–oligofructose (Synergy 1, Beneo-Orafti)	Oligofructose: 2–8 (4)Inulin: 10–60 (24)	16	2	NA	10 (5)	Healthy adults age 21–38 y, BMI 21.6 ± 0.99	↑ Postprandial GLP-1, PYY↔ Satiety, fasting GLP-1 and PYY↓ Hunger
(30)	R, DB, PC, CF	Cellulose	Inulin (Inulin HP, Beneo-Orafti)	≥23	20	6	≥3 (mean 6.3)	12	Nondiabetic adults with overweight/obesity, 60 ± 1 y, BMI 29.8 ± 0.9	↔ Fasting or postprandial GLP-1, PYY
(34)	R, SB, PC, CF	No ITF	Inulin (Inulin HP, Beneo-Orafti)	≥23	22.4	1	1	13	Healthy adults age 23 ± 4 y, BMI 22.1 ± 1.6	↔ Energy intake, macronutrient intake, hunger, fullness, prospective food consumption, desire to eat
(35)	R, SB, PC, P	Cellulose + Maltodextrin	Oligofructose (Beneo P95, Beneo-Orafti)	2–7	30	6	NA	22 (12)	Adults with overweight/obesity, age 20–49 y, BMI 30.33	↓ Hunger↔ Energy intake, fullness
(79)	R, DB, PC, P	Maltodextrin	Oligofructose (Raftilose P95, Beneo-Orafti)	2–7	21	12	NA	39 (21)	Adults with overweight/obesity, 40.4 y, BMI 30.1	↑ Postprandial PYY↓ Energy intake, postprandial ghrelin and leptin↔ Postprandial GLP-1, GIP
(81)	R, TB, PC, P	Maltodextrin	Oligofructose (Fructalose L92, Sensus)	2–10	1 wk @ 8, 11 wk @ 16	12	NA	55 (29)	Adults with overweight/obesity, 41 ± 12 y, BMI 29.4 ± 2.6	↓ Hunger, prospective food consumption↔ Energy intake
(51)	R, DB, PC, P	Placebo	Inulin (4 g) + Oligofructose (12 g)	NA	2 wk @ 8, 10 wk @ 16	12	NA	96 (26)	Adults with overweight/obesity, 39.8 y, BMI 31.5	↓ Hunger, desire to eat↔ Energy intake
(83)	R, DB, PC, CF	No ITF	Inulin (Raftiline HP-Gel, Orafti)	>23	11	5	8	20	Healthy adult men, 18.8 ± 0.7 y, BMI 22.8 ± 2.3	↑ Neurotensin, somatostatin↓ Gastric emptying rate (∼ somatostatin [+])↔ Corticotropin releasing factor
(120)	R, DB, PC, P	No ITF	ITF (Frutafit IQ, Sensus)	9–10	16	1	NA	36 (17)	Healthy adult women, 19.7 y	↑ Fullness↓ Desire to eat, hunger, prospective food consumption, food intake at lunch
(121)	R, DB, DR, PC, CF	Maltodextrin	Oligofructose (Fructalose L92, Sensus)	2–10	0, 10, 16	2	2	31	Healthy adults age 28 ± 3 y, BMI 24.8 ± 0.3	↑ Postprandial GLP-1, PYY (16 g/d dose)↓ Energy intake (16 g/d dose)
(40)	SGD	Habitual diet	ITF-rich vegetables (artichoke, garlic, salsify, shallot, leeks, scorzonera, onion, celery root)	NA	approx.15	2	3*	26	Healthy adults age 21.84 ± 0.39 y, BMI 22.29 ± 0.32	↑ Satiety, desire to eat ITF-vegetables↓ Desire to eat sweet/salty/fatty food

1 CF, crossover feeding; DR, dose ranging; DB, double-blinded; DP, degree of polymerization; GIP, glucose-dependent insulinotropic peptide; GLP-1, glucagon-like peptide 1; ITF, inulin-type fructans; MCF, multiple crossover feeding; NA, not applicable; P, parallel; PC, placebo-controlled; PYY, peptide YY; R, randomized; SB, single-blinded; SC, single-center; scFOS, short-chain fructooligosaccharides; SGD, single group-design; ˜ indicates a correlation; the parentheses indicate a positive (+) or negative (−) association.

These physiological mechanisms are only a part of the satiety story, which may be influenced by other behavioral, social, and other external factors that affect an individual's desire to eat (118). Therefore, subjective measures of appetite and satiety as well as objective measures of food and energy intake are crucial complementary measures to mechanistic markers. Hunger was generally decreased or lower following ITF intake (35, 40, 51, 81, 86, 119, 120), whereas fullness and satiety were increased or higher (40, 119, 120), though some studies reported no effect on hunger (27, 34) or satiety (34, 35, 86). Effects on energy intake were mixed (27, 34, 35, 51, 81), but several studies reported a decrease in or lower values of food or energy intake (79, 119–121). Additionally, a study by Hiel et al. reported that addition of ITF-rich vegetables (∼15 g/d ITF) to the diet increased the desire to eat ITF-rich vegetables and decreased the desire to eat sweet, salty, or fatty food (40). This suggests that inclusion of these ITF-rich foods promotes beneficial shifts in diet quality. These changes have the potential to beneficially impact weight management and body composition.

#### Body composition and energy balance

Obesity and body fat distribution, particularly visceral fat, increase risk of metabolic disease and CVD (122). Animal studies have demonstrated that intake of fermentable fibers, including inulin–oligofructose, can improve body composition by decreasing visceral and liver fat content (123) via mechanisms such as increased production of SCFAs and anorexigenic gut hormones, increased energy expenditure, and reduced energy availability (27, 29, 64). Most studies investigating the effects of ITF intake on body weight and body composition (**Table 8**) (26, 27, 35, 36, 51, 79, 81, 91) were conducted in adults who were overweight (BMI ≥25) or obese (BMI ≥30) (35, 36, 51, 79, 81, 91). Studies utilizing a hypocaloric diet protocol were excluded to isolate the effects of ITF on body composition. Only 1 study reported a reduction in body weight following ITF consumption (21 g/d) (79). The remaining studies reported no effect of ITF consumption on body weight or BMI (26, 27, 35, 36, 51, 81, 91). Additionally, no effects of ITF intake were observed on waist circumference or the waist-to-hip ratio (35, 36, 51, 81). More direct measures of body composition revealed some trending associations with ITF intake, including a decrease in fat mass (after 16 g/d of an inulin–oligofructose blend) (36) and fat-free mass (after 10 g/d inulin) (27). However, most studies reported no effect of ITF intake on measures of body composition, including fat mass, body fat percentage, total and regional adipose tissue, and lean mass (35, 51, 81). The lack of overall results on the effect of ITF intake on body weight and body composition suggest that ITF have little, if any, effect on these outcomes or that a longer study duration is needed to observe statistically significant changes.

**TABLE 8:** ITF effects on body composition and energy balance in healthy adults^1^

Ref.	Study design	Comparator	ITF type (trade name, manufacturer)	DP, range (mean)	ITF dosage, g/d	Intervention duration, wk	Washout duration, wk	N (*n*in ITF group)	Study population characteristics	Results
(26)	PC, CF, SB	Cereal (rice)	Inulin (Fibruline Instant, Cosucra)	2–60 (10)	9	4	4	12	Healthy adult men, 23.3 ± 0.5 y, BMI 25.7 ± 1.2	↔ Body weight
(27)	R, DB, PC, CF	No ITF	Inulin (Beneo HP, DKSH/Orafti)	≥23	10	1	2	21	Adults with overweight/obesity, 60 y, BMI 25–40	↔ Body weight, REE↓ Fat free mass (trend)
(35)	R, SB, PC, P	Cellulose + maltodextrin	Oligofructose (Beneo P95, Beneo-Orafti)	2–7	30	6	NA	22 (12)	Healthy adults with overweight and obesity, 20–49 y, BMI 30.33	↔ Body weight, W:H ratio, total and regional adipose tissue
(36)	R, DB, PC, P	Maltodextrin	Inulin–oligofructose (Synergy 1, Beneo-Orafti)	Oligofructose: 2–8 (4)Inulin: 10–60 (24)	16	12	NA	30 (15)	Adult women with obesity age 47.5 y, BMI 35.85	↔ BMI, W:H ratio↓ Fat mass (trend)*Bacteroides* & *Propionibacterium* ∼ fat mass %, fat mass/lean mass (+)
(79)	R, DB, PC, P	Maltodextrin	Oligofructose (Raftilose P95, Beneo-Orafti)	2–7	21	12	NA	39 (21)	Adults with overweight/obesity age 40.4 y, BMI 30.1	↓ Body weight
(81)	R, TB, PC, P	Maltodextrin	Oligofructose (Fructalose L92, Sensus)	2–10	1 wk @ 8, 11 wk @ 16	12	NA	55 (29)	Adults with overweight/obesity, 41 ± 12 y, BMI 29.4 ± 2.6	↔ Body weight, fat mass, lean mass, waist circumference
(51)	R, DB, PC, P	Placebo	Inulin (4 g) + scFOS (12 g)	NA	2 wk @ 8, 10 wk @ 16	12	NA	96 (26)	Adults with overweight/obesity, 39.8 y, BMI 31.5	↔ Body weight, fat mass, lean mass, body fat %, waist circumference
(91)	R, DB, PC, CF	Cellulose	Inulin (Fibruline DS2, Georg Breuer GmbH)	NA	30	1	1	16	Healthy adults age 40.5 ± 4.2 y, BMI 23.1 ± 1.0	↔ Body weight
(29)	R, P	Low nonstarch polysaccharide diet	Chicory inulin (Agro-industries, Recherches et Developpements Society)	NA	2 wk @ progressive intake, 12 d @ 50	3.7	NA	9	Healthy adult men, 21.5 + 2.5 y, BMI < 25	↔ EE (trend for increase), protein and lipid digestibility↓ Diet energy

1 CF, crossover feeding; DR, dose ranging; DB, double-blinded; DP, degree of polymerization; EE, energy expenditure; ITF, inulin-type fructans; NA, not applicable; P, parallel; PC, placebo-controlled; R, randomized; REE, resting energy expenditure; SB, single-blinded; SC, single-center; scFOS, short-chain fructooligosaccharides; SGD, single group-design; W:H ratio, waist-to-hip ratio; ˜ indicates a correlation; the parentheses indicate a positive (+) or negative (−) association.

Only Dewulf et al. investigated the correlation between the fecal microbiota and changes in body weight and body composition following ITF consumption (36). In that study, members of the genera *Propionibacterium* and *Bacteroides*, which were decreased following intake of an inulin–oligofructose blend (16 g/d), were positively associated with fat mass percentage and the fat mass to lean mass ratio (36).

Two studies have investigated the effect of ITF intake on energy expenditure (27, 29). Byrne et al. reported no effect of inulin (10 g/d) on resting energy expenditure in healthy adults (27). Castiglia-Delavaud et al. reported numerically higher total energy expenditure following inulin intake (50 g/d) compared with control, but this was not statistically significant (29). Castiglia-Delavaud et al. also investigated the effect of ITF inclusion on metabolizable energy and nutrient utilization of the diet. The metabolizable energy of the diet was lower with ITF than with control consumption, but this had no effect on protein or lipid utilization (29). No correlations between the fecal microbiota and changes in energy expenditure following ITF intake were investigated.

### Conclusions and Future Directions

The objective of the current review was to summarize the potential of ITF to act as a prebiotic in adults according to the parameters listed in the published ISAPP scientific definition. According to this definition, a prebiotic is selectively utilized by the gastrointestinal microbiota and confers a health benefit (5). Evidence suggests that ITF have a strong bifidogenic effects on the human fecal microbiota and promote the growth and activity of other bacteria with known beneficial health effects, such as *Lactobacillus* and *F. prausnitzii*. These effects on the intestinal microbiota contribute to benefits on human health, including improved intestinal barrier function, improved laxation, increased insulin sensitivity, decreased blood triglycerides and an improved lipid profile, increased calcium and magnesium absorption, and increased satiety. Evidence is inconclusive or limited for the effects of ITF on intestinal microbiota function and SCFA production, bile acid concentrations, insulin and incretin hormone concentrations, cholesterol and lipoprotein composition, bone health and absorption of iron or zinc, inflammation, objective markers of appetite and satiety, and body weight and body composition.

Future research on this topic using -omics technologies is likely to help elucidate mechanisms by which the intestinal microbiota mediate or modify these effects and the impact of factors intrinsic to ITF (e.g., chain length, source) and individuals (e.g., age, health status). Though some evidence from studies in the current review suggests differential effects of ITF on *Bifidobacterium*, SCFAs, lipids, and mineral absorption based on differences in chain length, lack of direct comparisons and detailed reporting of ITF characteristics make it difficult to draw conclusions on the effect of chain length on the intestinal microbiome. Thus, given the scarcity of studies comparing ITF types based on intrinsic factors such as source or chain length, further research is warranted. This research should consider individual factors such as baseline intestinal microbiota composition that could confound the effects of these variables.

Individual factors such as baseline intestinal microbiota composition, habitual fiber intake, and age or health status (e.g., prediabetic subtype, lipid levels, bone density, etc.) may also contribute to interindividual variability in the effects of ITF intake on health outcomes (41, 87, 88). Results of studies in the current review suggest that individuals’ baseline fecal microbiota composition, particularly abundance of *Bifidobacterium*, may modify the effects of ITF on the microbiota. However, the lack of studies investigating the correlations between baseline fecal microbiota composition and health outcomes following ITF consumption make it difficult to draw conclusions. Although correlations between the fecal microbiota and other individual factors, metabolite production, and subsequent health effects may suggest plausible relationships, establishing causality and mechanisms of action remain challenges within the field of human microbiota research. Multitiered approaches that use a combination of human, animal, and in vitro models, as well as the continuing development of methods of noninvasive sampling and biomarker discovery, will improve our ability to establish causal relationships between ITF intake, the intestinal microbiota, and health outcomes. This research will facilitate the formulation of personalized dietary recommendations and strategies to improve human health using prebiotics such as ITF.

## Notes

This work was partially supported by General Mills.

Author disclosures: The authors report no conflicts of interest.

Abbreviations used: BCFA, branched-chain fatty acid; CINAHL, Cumulative Index to Nursing and Allied Health Literature; CVD, cardiovascular disease; DP, degree of polymerization; FOS, fructooligosaccharide; GIP, glucose-dependent insulinotropic peptide; GLP-1, glucagon-like peptide-1; HbA1c, hemoglobin A1c; IR, insulin resistance; ISAPP, International Scientific Association for Probiotics and Prebiotics; ITF, inulin-type fructans; Lp(a), lipoprotein(a); OGTT, oral glucose tolerance test; PAI-1, plasminogen activator inhibitor-1; PRISMA, Preferred Reporting Items for Systematic Reviews and Meta-analyses; PTH, parathyroid hormone; PYY, peptide YY; scFOS, short-chain fructooligosaccharides; VAS, visual analogue scale.
